# Theory of mind in mild cognitive impairment and Parkinson’s disease: The role of memory impairment

**DOI:** 10.3758/s13415-023-01142-z

**Published:** 2023-12-04

**Authors:** Gianpaolo Maggi, Chiara Giacobbe, Carmine Vitale, Marianna Amboni, Ignacio Obeso, Gabriella Santangelo

**Affiliations:** 1 Department of Psychology, University of Campania “Luigi Vanvitelli,” Viale Ellittico, 31, 81100 Caserta, Italy; 2 Institute of Diagnosis and Health, IDC-Hermitage Capodimonte, Naples, Italy; 3 Department of Motor Sciences and Wellness, University “Parthenope, Naples, Italy; 4 Department of Medicine, Surgery and Dentistry, University of Salerno, Salerno, Italy; 5 HM Hospitales – Centro Integral de Neurociencias AC HM CINAC, Hospital Universitario HM Puerta del Sur, HM Hospitales, Avda. Carlos V, 70. 28938, Móstoles, Madrid, Spain; 6 Department of Psychobiology and Methods on Behavioural Sciences, Complutense University of Madrid, Madrid, Spain

**Keywords:** Parkinson’s disease, Social cognition, Theory of mind, Mild cognitive impairment, Memory

## Abstract

**Background:**

Social cognition is impaired in Parkinson’s disease (PD). Whether social cognitive impairment (iSC) is a by-product of the underlying cognitive deficits in PD or a process independent of cognitive status is unknown. To this end, the present study was designed to investigate the weight of specific cognitive deficits in social cognition, considering different mild cognitive impairment subtypes of PD (PD-MCI).

**Methods:**

Fifty-eight PD patients underwent a neuropsychological battery assessing executive functions, memory, language, and visuospatial domains, together with social cognitive tests focused on theory of mind (ToM). Patients were divided into subgroups according to their clinical cognitive status: amnestic PD-MCI (PD-aMCI, *n* = 18), non-amnestic PD-MCI (PD-naMCI, *n* = 16), and cognitively unimpaired (PD-CU, *n* = 24). Composite scores for cognitive and social domains were computed to perform mediation analyses.

**Results:**

Memory and language impairments mediated the effect of executive functioning in social cognitive deficits in PD patients. Dividing by MCI subgroups, iSC occurred more frequently in PD-aMCI (77.8%) than in PD-naMCI (18.8%) and PD-CU (8.3%). Moreover, PD-aMCI performed worse than PD-CU in all social cognitive measures, whereas PD-naMCI performed worse than PD-CU in only one subtype of the affective and cognitive ToM tests.

**Conclusions:**

Our findings suggest that ToM impairment in PD can be explained by memory dysfunction that mediates executive control. ToM downsides in the amnesic forms of PD-MCI may suggest that subtle changes in social cognition could partly explain future transitions into dementia. Hence, the evaluation of social cognition in PD is critical to characterize a possible behavioral marker of cognitive decline.

**Supplementary Information:**

The online version contains supplementary material available at 10.3758/s13415-023-01142-z.

## Introduction

Social cognition is a crucial neurocognitive capacity that determines how individuals recognize, understand, and respond to social cues (Henry et al., 2016). Failures of social cognition, such as poor empathy, impaired theory of mind (ToM), altered social perception, and abnormal social behavior, are prominent in several neurological disorders and represent critical predictors of functional outcomes and quality of life due to their impact on the ability to create and maintain successful interpersonal relationships (Henry et al., 2016). Indeed, this neurocognitive domain encompasses four broad components: 1) ToM, which refers to the ability to understand the mental states of others; 2) empathy, defined as the capacity to engage with and share others’ emotions; 3) social perception, which refers to the ability to interpret and respond to social cues; and 4) social behaviour, described as the capacity to behave appropriately in social situations (Henry et al., 2016; Michaelian et al., 2019).

More specifically, ToM is a multidimensional construct that can be differentiated into two subcomponents: a cognitive subcomponent, defined as the capacity to understand the intentions, beliefs, and desires of others, and an affective subcomponent that consists of understanding others’ emotions, feelings, and affective states (Kalbe et al., 2010a; Shamay-Tsoory et al., 2004; Shamay-Tsoory & Aharon-Peretz, 2007). Social perceptual deficits, such as problems in recognizing basic emotional cues may disrupt the ability to correctly understand others’ mental states; thus, the ability to recognize emotions seems to represent the first automatic level of intention attribution and mindreading (Coricelli, 2005; Phillips et al., 2002; Sagliano et al., 2020), needed across several social interactions. Hence, the correct use of social cognition determines successful adaptive behaviour to contextual demands to enhance interpersonal relationships at different levels.

Adaptive use of social cognition is supported by specific cognitive functions, such as executive function, language, memory, and visuospatial abilities. Evidence of a close relationship between ToM abilities and executive functioning when evaluating false-belief and perspective-taking tasks has been found in healthy subjects (Saltzman et al., 2000). Indeed, social cognition and language probably build an evolutionary cycle in which the development of one feeds the other (Fitch et al., 2010), given that language skills support several key abilities needed for inter-individual communication (e.g., exchanging social information, reasoning about others’ mental states, and expressing and recognizing communicative intentions). Also, memory functions seem to contribute to social cognition feeding a “social knowledge”—a type of semantic memory regarding information about the retrieval of appropriate social words, social behaviors, and social entities gained throughout experience (Olson et al., 2013). Importantly, correct retrieval of the needed social words (a language-related process) is frequently used to take others’ perspectives and behave in a social manner (Olson et al., 2013). It has been argued that preserved episodic memory could be crucial for adaptive ToM mediated by the long-term recollection of relevant social information, a key mechanism in the understanding of others’ beliefs and feelings needed to interpret social situations (Laillier et al., 2019). Hence, adequate computation along various cognitive functions ultimately determines the quality of social cognition.

Several of the above cognitive domains associated with social cognitive processes are impaired in Parkinson’s disease (PD), which may explain the more severe ToM dysfunction in PD patients in front of less severe deficits in empathy and social perception, as revealed by the effect sizes of recent meta-analytic studies (Coundouris et al., 2019, 2020). Neuropathological grounds for executive impairments in PD include dopaminergic denervation, which initially affects caudal putamen (Pineda-Pardo et al., 2022), leading to frontal changes in the dorsolateral prefrontal cortex (DLPFC) and impairments in set-shifting and inhibition (Obeso et al., 2011; Sawada et al., 2012). As neurodegeneration spreads to other frontal areas, such as the orbitofrontal cortex (OFC) and medial frontal cortex (mPFC), as well as some portions of the limbic network (Díez-Cirarda et al., 2015; Ibarretxe-Bilbao et al., 2009; Kalbe et al., 2010b; Péron et al., 2010), manifestations also involve affective processing, decision-making, memory, and language difficulties (Banwinkler et al., 2022). Importantly, functional changes in DLPFC and ventromedial PFC contribute to social cognitive impairments observed in PD (Díez-Cirarda et al., 2015; Ibarretxe-Bilbao et al., 2009; Kalbe et al., 2010a; Péron et al., 2009), especially in emotion recognition (Coundouris et al., 2019) and ToM (Bora et al., 2015; Coundouris et al., 2020). Thus, PD represents an ideal model to test predictions on which cognitive subdomains may impact the adequate execution of social cognition differently. Notably, whether social cognitive deficits in PD are a by-product of general cognitive difficulties rather than specific isolated deficits remains unknown.

In our recent meta-analysis, we found that ToM deficits in PD were related to executive and language impairments (Maggi et al., 2022a). Dissociated patterns of neuropsychological correlates emerged between different ToM subcomponents: cognitive ToM abilities widely related to all executive functions, while affective ToM was specifically linked to some executive functions, such as cognitive flexibility and decision-making (Maggi et al., 2022a, b). Other executive functions have been linked to ToM deficits in PD, such as working memory (Enrici et al., 2017; Kosutzka et al., 2019), flexibility (Díez-Cirarda et al., 2015; Kosutzka et al., 2019), and inhibition problems (Foley et al., 2019). Language processing, which is impaired in PD due to basal ganglia dysfunctions (Chenery et al., 2008; MacOir et al., 2013), also might contribute to ToM alterations, because it provides a supporting structure, together with executive mechanisms, for reasoning about others’ emotional and mental states critical in some mentalizing operations during social cognitions, such as detection of emotional prosody, humor, and sarcasm (Bodden et al., 2010; Maggi et al., 2022a, b). Also other neuropsychological domains may contribute to social cognition deficits in PD, such as memory (Alonso-Recio et al., 2021; Enrici et al., 2017) and visuospatial abilities (Kosutzka et al., 2019; McKinlay et al., 2013). In summary, an overall cognitive spectrum of interrelated functions subserves adaptive social cognition, an impaired function in PD, with special emphasis on executive control (Maggi et al., 2022a, b).

The aforementioned studies included PD patients without overt cognitive decline. However, the implication of mild cognitive impairment (MCI) as a confounding factor in ToM-related cognitive mechanisms in PD remains uncertain. Progressive impairment in social cognition abilities in advanced disease stages with concomitant presence of MCI has been reported (Alonso-Recio et al., 2021; Anderson et al., 2013), whereas other studies have revealed social cognitive impairment in early PD patients with preserved cognitive abilities (Czernecki et al., 2021; Dodich et al., 2022), suggesting the existence of an isolated social cognitive impairment. Thus, the exact nature of social cognitive impairment in PD, as well as the specific weight of each cognitive subdomain in its manifestation, remains unclear. Importantly, no study has explored the occurrence of social cognitive impairment in different clinical subtypes (i.e., amnestic and non-amnestic) of PD-MCI, which may impact social cognitive processes in PD differently.

The present study aimed to investigate whether social cognitive deficits, especially ToM impairment, in PD are a by-product of several cognitive domains, and how they relate to different PD-MCI profiles (i.e., amnestic and non-amnestic). Given that impairments in executive control seem to explain ToM impairment in PD (Maggi et al., 2022a, b), we first explored the role of cognitive impairments (i.e., memory, language, and visuospatial abilities) as mediators of the relationship between executive dysfunctions and social cognition in PD. We then examined whether and how the occurrence and extent of social cognitive impairment are associated with specific PD-MCI profiles (i.e., amnestic and non-amnestic) compared with patients with preserved cognition.

## Methods

### Participants

We screened consecutive PD outpatients referred to the Movement Disorders Unit of the IDC-Hermitage Capodimonte (Naples, Italy). Fifty-eight PD patients (38 males and 20 females; mean age = 64.57, standard deviation [SD] = 8.68) were included in the present study. PD patients were included in the study if the following inclusion criteria were met: 1) diagnosis of idiopathic PD according to the UK Brain Bank diagnostic criteria (Hughes et al., 1992); 2) absence of dementia, evaluated by both MDS criteria (Emre et al., 2007) and means of the Italian version of the Montreal Cognitive Assessment (MoCA > 15.5 (Santangelo et al., 2015)]); 3) absence of neurological conditions other than PD; and 4) absence of major depressive symptomatology according to the clinical diagnostic criteria of DSM-V after an initial screening based on the Italian cutoff of the Beck Depression Inventory-II (BDI-II) (Maggi et al., 2023).

We collected demographic (i.e., age, sex, educational level) and clinical data as the disease duration (years from diagnosis), medication (Levodopa Equivalent Daily Dose; LEDD), stage of the disease and severity of motor symptoms assessed by Hoehn & Yahr staging system (H&Y) and part III of Unified Parkinson’s Disease Rating Scale (UPDRS-III), respectively, and neuropsychiatric symptoms using the BDI-II (Maggi et al., 2023)—the Parkinson Anxiety Scale and the Dimensional Apathy Scale (Aiello et al., 2022). All participants provided their informed written consent to participate before starting the study, which was approved by the Local Ethics Committee and performed according to the ethical standards laid down in the Declaration of Helsinki and its later amendments.

### Neuropsychological evaluation

#### Social cognition

Social cognition assessment consisted of the evaluation of both the cognitive and emotional domains of theory of mind processes. The Reading the Mind in the Eyes Test (RMET) is a widely used assessment tool for social cognitive functions, because it evaluates how individuals recognize complex emotional expressions from the eye region of adult faces. Although some evidence has demonstrated that it is more specific for the evaluation of more automatic and implicit processes, such as emotion recognition (Baron-Cohen et al., 1997; Oakley et al., 2016; Quesque & Rossetti, 2020), the RMET was conceived and currently employed as an Affective ToM measure. The Italian version of the RMET consists of 36 photographs of the eye part of the face, accompanied by four words depicting emotional states placed around the pictures. Subjects are asked to choose the word that best describes the emotional state of each photograph. For this test, we calculated the sum of the correct responses.

To assess the affective component of ToM, we also used the modified Italian version of the Emotion Attribution Task (EAT; Blair & Cipolotti, 2000), which consists of 35 short stories eliciting the attribution of several emotions (i.e., sadness, fear, embarrassment, disgust, happiness, anger, and envy). Subjects must understand how the characters may feel in a particular situation and choose the closest feeling. The total score reflects the number of emotions correctly attributed.

Otherwise, the Strange Stories (ATT) developed by Happé (1994) was used as a measure of cognitive ToM. The test comprises several short stories depicting naturalistic situations in which two or more characters interact in familiar or social contexts. These interactions include white lies, double bluff, mistakes, and persuasion. The subject is asked to explain why the characters say something or behave as they do (Maggi et al., 2022a, b). We calculated the sum of stories correctly understood and interpreted.

The Theory of Mind Picture Stories Task (TMPS) developed by Brüne (2003) evaluates the ability to infer others’ mental states (cognitive ToM) and predict their behavior on different levels of intentionality. Using a series of six cartoon picture stories, different scenarios are presented that show reciprocity, deception, and cheating. Participants are then asked to order the four cartoon cards in a logical sequence of events and describe the overall story shown in the pictures. Finally, they are asked questions to examine their comprehension of several aspects of the stories regarding reciprocity, deception, cheating, and reality. We calculated the total accuracy score as a result of general story comprehension and several subscores reflecting the comprehension of first-order, second-order, and third-order false beliefs and other comprehension variables regarding reciprocity, deception, cheating detection, and reality. We transformed the raw scores of above tests into z-scores (calculated on sample data) and computed the sum of these scores to create a social cognition composite score. Based on general neuropsychological standards in PD (Goldman et al., 2015), patients reporting a composite score in social cognition tests <1.5 SD of the group average were classified as presenting social cognitive impairment (PD-iSC). Meanwhile, patients reporting a score >1.5 SD were classified as unimpaired social cognitive impairment (PD-uSC).

#### Derivation of composite domain scores

We calculated the composite score for each neuropsychological domain by summing the z-scores (obtained from normative adjusted scores) of each cognitive domain. Particularly, we categorized a memory composite score by integrating the delayed recall of RAVLT together with the delayed recall of a short story; the visuospatial and constructional abilities composite score was created with BJLOT and the Constructional Apraxia test; language composite score was calculated by using the Nouns and Verb Naming tasks; the executive control composite score consisted of tests evaluating both executive functions and attention/working memory (i.e., the Color reading task and Color-word Interference task of the Stroop test, the phonological verbal fluency task, and part A and B-A scores of the Trail Making Test [TMT]). We employed this relatively broad and inclusive measure of executive control based on previous literature supporting the multifactorial nature of executive functions (Diamond, 2013; Enticott & Ogloff, 2006; Friedman & Miyake, 2004) and demonstrating the involvement of this wide domain in ToM processes (Maggi et al., 2022a, b). Furthermore, these four cognitive composite measures (i.e., Memory, Visuospatial and constructional abilities, Language, and Executive control) were validated by proposing and testing a factor model. The references for the cognitive tests employed in this study are reported in Supplemental Material 1.

#### MCI subtypes classification

To detect the occurrence of PD-MCI using Level II criteria (Litvan et al., 2012), patients underwent a complete neuropsychological assessment exploring five cognitive domains (using at least two tests in each domain) including: 1) Attention/working memory/processing speed, evaluated using the Color task of the Stroop test and part A of the Trail Making Test (TMT); 2) Executive functions assessed by the Color-word Interference task of the Stroop test, phonological verbal fluency task, and part B-A of the TMT; 3) Visuospatial and constructional abilities evaluated by the Benton Judgment of Line Orientation (BJLOT) and Constructional Apraxia test; 4) Memory assessed by the delayed recall of the Rey Auditory Verbal Learning Test (RAVLT) and the delayed recall of a short story “Anna Pesenti”; and 5) language assessed by the Nouns and Verb Naming tasks (Neuropsychological Exam for Aphasia).

To evaluate whether patients’ performance significantly diverged from normal performance, we converted the adjusted scores of each neuropsychological measure into *z*-scores using available normative data. Patients reporting performance 1.5 SDs below the appropriate norms on at least two tests were classified as PD-MCI following the criteria proposed by Litvan et al. (2012). Subsequently, PD-MCI patients were classified as amnestic and non-amnestic based on the presence of memory impairment, following a subdivision reported in previous studies (Chung et al., 2019; Devignes et al., 2022): participants with MCI who demonstrated a non-amnesic cognitive profile (PD-naMCI) and those showing an amnesic profile in at least one of the abovementioned memory tests (PD-aMCI).

### Statistical analysis

A power estimation was carried out to determine the sample size required for our analyses, establishing an alpha level of 0.05, and a large effect size of η^2^ = 0.50 (Faul et al., 2007).

We conducted confirmatory factor analysis (CFA) in AMOS to test the hypothesized factor model for cognitive composite scores. Model fit was evaluated by the Confirmatory Fit Index (CFI) and the Tucker-Lewis index (TLI), where values >0.95 for both indicate an excellent fit. In addition, the Root Mean Square Error of Approximation (RMSEA) was used with values <0.08 showing adequate fit, and values <0.05 were an excellent fit to the data.

Subsequently, to examine whether and how cognitive domains mediated the relationship between Executive control and ToM processes in the whole sample, we performed several parallel mediation analyses, entering ToM scores as dependent variables, Executive control composite score as predictor, and Memory, Visuospatial, and constructional abilities, and Language composite scores as parallel mediators. Further mediation models were performed with single executive tests as predictors to explore the differential contributions of executive subdomains. We used a bootstrapping procedure with 5000 samples and replacement from the full sample to construct bias-corrected 95% confidence intervals (CI) (LL = lower level of confidence interval, UL = upper level of confidence interval). Mediation models were performed by using SPSS Macro PROCESS (Preacher & Hayes, 2008).

Moreover, the main variables of interest were compared between the three different types of cognitive subgroups: PD controls (PD-CU), PD-aMCI, and PD-naMCI. Then, a subdivision based on social cognition compared those patients with impairments (PD-iSC) against those with unimpaired social cognition (PD-uSC). We evaluated the association between the co-occurrence of MCI and iSC using the chi-squared (χ^2^) test to compare proportions in a two-by-three contingency table. Pairwise comparisons using Kruskal-Wallis tests and Bonferroni corrections were performed to determine the statistically significant differences between the three MCI groups.

To control for the possible effect of clinical variables on social cognitive tests and cognitive composite scores, we performed several multiple regression analyses, entering scores on social cognitive tests as dependent variables and clinical variables, such as disease duration, UPDRS-III scores, LEDD, and BDI-II scores, as predictors.

The significance level was set at α = .05, and all statistical analyses were performed by using SPSS Statistic 26.0 (IBM, 2019). Power analysis was performed by using G*Power 3.1 (Faul et al., 2007).

## Results

Power analysis indicated that a total sample size of 42 patients was required to reach statistical significance with power (1−β) set at 0.80, an estimated medium effect size of 0.5, and a *p* value of 0.05 (two-tailed). The final sample comprised of 58 patients. Our participants reported an average level of education (mean = 11.64, SD = 4.44) and a mean disease duration of 10.22 years (SD = 5.98).

### Mediation analysis: Cognitive subdomains links with executive dysfunction and social cognition

Four mediation models were designed to test the possible mediation effects of Memory, Visuospatial and constructional abilities, and Language scores on the relationship between Executive control and the SC tests. An excellent model fit (CFI = 0.981, TLI = 0.967, RMSEA = 0.035) for the proposed model solution of cognitive composite scores (i.e., Executive control, Memory, Visuospatial and constructional abilities, and Language) was demonstrated by the CFA.

**Fig. 1:**
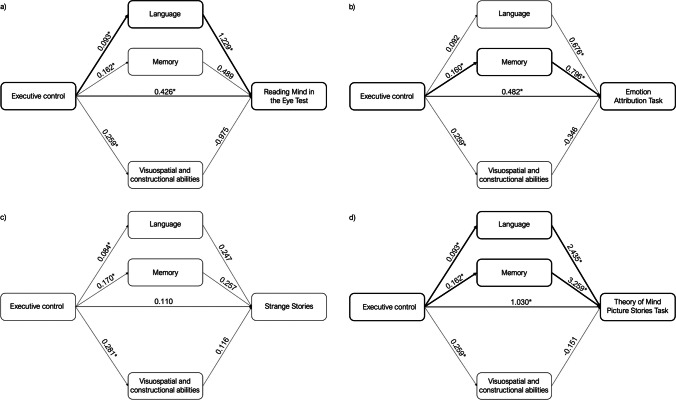
Mediation effects (represented by bold lines) of neurocognitive domains in the relationship between Executive control and performance on social cognitive tests. *p < 0.05

Altered Executive control was associated with worse scores on Language (*B* = 0.093; *p* = 0.050), Memory (*B* = 0.162; *p* = 0.001), and Visuospatial and constructional abilities (*B* = 0.259; *p* < 0.001) domains. Subsequently, poorer performance on Executive control (*B* = 0.426; *p* = 0.012) and Language (*B* = 1.229; *p* = 0.006) were associated with worse scores on the RMET (emotion recognition/affective ToM). Examination of the indirect effects using the bootstrap-generated bias-corrected CI approach demonstrated the indirect effect of Executive control on RMET performance through Language domain (Estimate effect: 0.114; 95% CI 0.002–0.282) (Fig. 1a).

This result was confirmed when using Inhibition (evaluated by the Interference task of the Stroop test) as predictor, both Memory and Language emerged as mediators entering Processing speed (measured by part A of the TMT) as the independent variable, whereas no significant mediation effect was found by using other executive tests as predictors (Supplementary Material 2).

In addition, impaired Executive control (*B* = 0.482; *p* = 0.001) and Memory (*B* = 0.796; *p* = 0.027) were associated with poorer performance on the EAT (affective ToM). The examination of the indirect effects showed a significant mediation effect of Memory domain (Estimate effect, 0.127; 95% CI 0.029–0.236) on the relationship between Executive control and EAT (Fig. 1b). The same result was confirmed when single executive tests were used as predictors (Supplementary Material 3).

Conversely, no association between cognitive domains and Strange Stories scores emerged. We did not find any indirect effect, but the total effect, the sum of indirect and direct effects, of Executive control on Strange Stories was significant (Estimate effect, 0.207; 95% CI 0.051–0.363) (Fig. 1c). The absence of significant mediation effects was confirmed by entering single executive tests as predictors (Supplementary Material 4).

Finally, worse cognitive TOM scores (TMPS) were related to poorer Executive control (*B* = 1.030; *p* < 0.001), altered Memory abilities (*B* = 3.259; *p* < 0.001), and impaired Language (*B* = 2.435; *p* = 0.003). The examination of the indirect effects showed two parallel indirect effects of Executive control on TMPS through both Memory (Estimate effect, 0.528; 95% CI 0.195–0.947) and Language (Estimate effect, 0.227; 95% CI 0.017–0.541) domains (Fig. 1d). The mediation effect of Memory was confirmed when using single executive tests as predictors, whereas Language contribution was significant when entering part A and B-A scores of the TMT and Interference task of the Stroop test (Supplementary Material 5).

### Clinical and neuropsychological results in mci subtypes

According to the MDS task-force criteria (Litvan et al., 2012), 18 PD patients were classified as PD-aMCI (all multiple-domain MCI patients), 16 as PD-naMCI (six single-domain MCI patients), and 24 as cognitively unimpaired PD. Demographic and clinical details of the amnesic and non-amnesic PD patients are reported in Table 1.

**Table 1:** Comparison between PD groups on socio-demographical, clinical, neuropsychiatric and neuropsychological variables

	PD-CU (*n*=24) A	PD-aMCI (*n*=18) B	PD-naMCI (*n*=16) C		
	Mean **±** SD	Mean **±** SD	Mean **±** SD	χ^2^	*p*
Age	62.08 **±** 8.45	62.78 **±** 8.02	70.31 **±** 7.43	10.198	**0.006** **C>A, B**
Educational level (ys)	12.33 **±** 3.83	11.28 **±** 4.72	11 **±** 5.08	0.772	0.680
Gender (M:F)	16:8	10:8	12:4	1.442	0.486
Clinical Variables					
Disease duration (ys)	8.88 **±** 4.87	13.28 **±** 6.99	8.81 **±** 5.27	4.852	0.088
H&Y	2.00 **±** 0.36	2.62 **±** 0.76	2.53 **±** 0.67	13.347	**0.001** **B, C>A**
UPDRS-III score	11.71 **±** 5.5	14.82 **±** 7.87	21.93 **±** 8.55	12.93	**0.002** **C>A**
LEDD	673 **±** 357.26	948.76 **±** 481.69	615.93 **±** 427.12	5.559	0.062
Neuropsychiatric Variables					
BDI-II	7.3 **±** 9.31	9.83 **±** 6.79	4.07 **±** 3.86	8.992	**0.011** **B>C**
PAS	10.43 **±** 9.88	11.33 **±** 9.3	9.47 **±** 7.2	0.195	0.907
DAS	21.96 **±** 11.89	21.28 **±** 9.48	25.4 **±** 7.86	2.683	0.261
Neuropsychological Variables					
MoCA	24.3 **±** 3.05	17.67 **±** 3.72	18.5 **±** 4.11	24.223	**<0.001** **B, C<A**
RAVLT- immediate recall	40.42 **±** 13.1	28.44 **±** 12.21	28.94 **±** 11.85	10.282	**0.006** **B<A**
RAVLT – delayed recall	8.62 **±** 3.31	5.44 **±** 3.31	6.94 **±** 2.86	10.886	**0.004** **B<A**
Short story – delayed recall	13.25 **±** 4.21	6.56 **±** 3.69	10.57 **±** 4.36	19.175	**<0.001** **B<A**
Constructional apraxia test	12.61 **±** 1.34	10.78 **±** 2.21	11.07 **±** 2.19	8.751	**0.013** **B<A**
BJLOT	22.29 **±** 3.18	14.11 **±** 5.52	15.62 **±** 5.28	26.144	**<0.001** **B, C<A**
Object naming	9.91 **±** 0.29	9.83 **±** 0.38	9.62 **±** 0.5	5.054	0.080
Verb naming	9.52 **±** 0.51	8.11 **±** 0.76	8.44 **±** 1.41	22.174	**<0.001** **B, C<A**
TMT A	40.54 **±** 17.04	102.39 **±** 67.39	80.5 **±** 41.25	23.897	**<0.001** **B, C>A**
TMT B-A	75.83 **±** 43.6	215.35 **±** 120.41	231.87 **±** 121.55	24.070	**<0.001** **B, C>A**
Stroop Test - Colour	41.62 **±** 9.28	29.44 **±** 8.29	30.75 **±** 4.58	15.656	**<0.001** **B, C<A**
Stroop Test – Interference	20.04 **±** 7.67	9.44 **±** 5.7	8.5 **±** 4.72	21.917	**<0.001** **B, C<A**
Phonological fluency	33.25 **±** 8.99	26.39 **±** 9.36	24.62 **±** 9.45	9.201	**0.010** **B, C<A**

PD= Parkinson’s Disease; MCI= Mild Cognitive Impairment; PD-aMCI= PD patients with amnestic MCI; PD-naMCI= PD patients with non-amnestic MCI; SD= Standard Deviation; ys= years; M= Male; F= Female; H&Y= Hoehn and Yahr staging system; UPDRS= Unified Parkinson’s Disease Rating Scale; LEDD= Levodopa Equivalent Daily Dose; BDI-II= Beck Depression Inventory-II; PAS= Parkinson Anxiety Scale; DAS= Dimensional Apathy Scale; MoCA= Montreal Cognitive Assessment; RAVLT= Rey Auditory Verbal Learning Test; BJLOT= Benton Judgement of Line Orientation; TMT= Trail Making Test

Bold *p* values represent the statistically significant results

The PD-naMCI patients were older than PD-CU (*p* = 0.009) and PD-aMCI (*p* = 0.026) patients, whereas no significant difference emerged on education level (Table 1). The clinical variables showed that groups differed on disease stage (based on H&Y scores), with PD-aMCI and PD-naMCI groups reporting more advanced PD stage than PD-CU (Table 1). As for UPDRS-III score, we found that PD-naMCI patients reported worse motor disabilities than PD-CU patients (*p* = 0.001). Conversely, no significant differences were observed on disease duration and LEDD between groups.

The neuropsychiatric condition revealed group differences on depressive symptoms with PD-aMCI scoring higher on the BDI-II compared to PD-naMCI group (*p* = 0.014), whereas no significant difference was found on anxiety and apathetic symptoms (Table 1).

As expected, PD-aMCI and PD-naMCI groups showed lower global cognitive functioning (based on MoCA score) than PD-CU (*p* < 0.001). Regarding memory domain, groups differed on both Immediate and Delayed recall of RAVLT with PD-aMCI reporting worse performance than PD-CU (*p* = 0.008 and *p* = 0.003, respectively) and on the Delayed recall of a short story where PD-aMCI obtained lower scores compared to PD-CU (*p* < 0.001) and PD-naMCI (*p* = 0.049). We found significant differences between groups on visuospatial and constructional tests with PD-aMCI performing worse on Constructional Apraxia test compared with PD-CU (*p* = 0.020) and PD-aMCI and PD-naMCI reporting lower scores on BJLOT than PD-CU (*p* < 0.001). Groups differed on language skills in the Verb Naming test where PD-aMCI and PD-naMCI produced poorer scores than PD-CU (*p* < 0.001 and *p* = 0.012, respectively), whereas no difference emerged on Object Naming test (Table 1). Finally, significant differences emerged on tests that evaluated executive and attention domain, such as part A and B-A of the TMT, Color and Interference tasks of the Stroop test, and phonological fluency where PD-aMCI and PD-naMCI obtained worse performance compared with PD-CU (Table 1).

### Social cognition and MCI subtypes

**Fig. 2:**
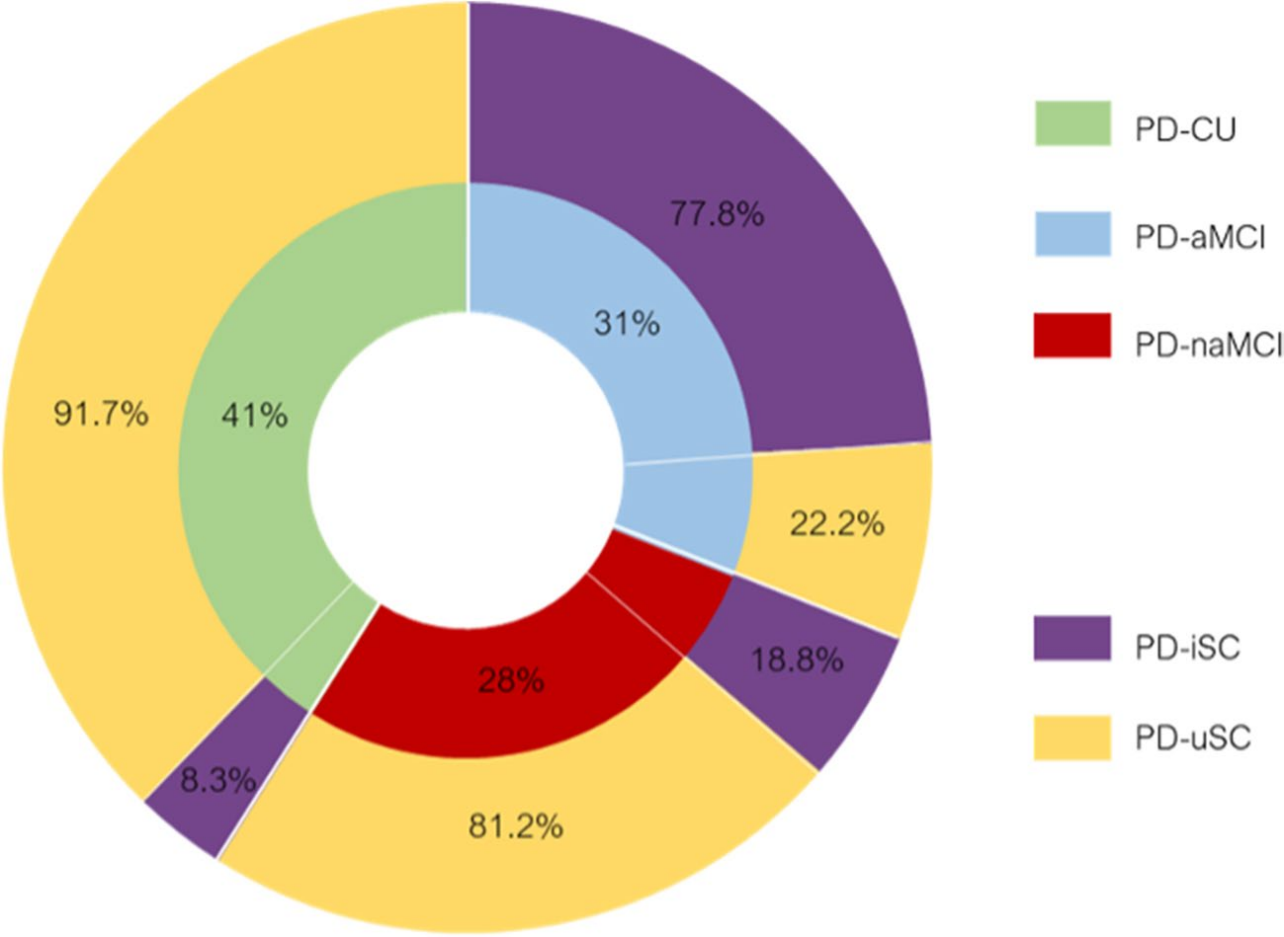
Percentages of groups distribution according to the presence of amnestic mild cognitive impairment (PD-aMCI), nonamnestic mild cognitive impairment (PD-naMCI), and preserved cognitive functions (PD-CU). Frequencies for social cognitive impairment (PD-iSC) and preserved social cognitive functioning (PD-uSC) were described for all groups

Thirty-nine PD patients did not present social cognitive impairment and were classified as PD-uSC, whereas 19 patients were assigned to PD-iSC group. Considering the whole sample, chi-squared tests of independence showed a significant relationship between the occurrence of social cognitive impairment within the three groups (degrees of freedom [*df*] = 2; χ^2^ = 24.487; *p* < 0.001). In particular, we observed more social impairments in the PD-aMCI group (*n* = 14, 77.8%) than in the PD-CU (*n* = 2, 8.3%) and PD-naMCI (*n* = 3, 18.8%) groups, whereas preserved social cognition was higher in the PD-CU group (*n* = 22, 91.7%) and PD-naMCI (*n* = 13, 81.2%) groups than in the PD-aMCI group (*n* = 4, 22.2%) (Fig. 2).

**Fig. 3:**
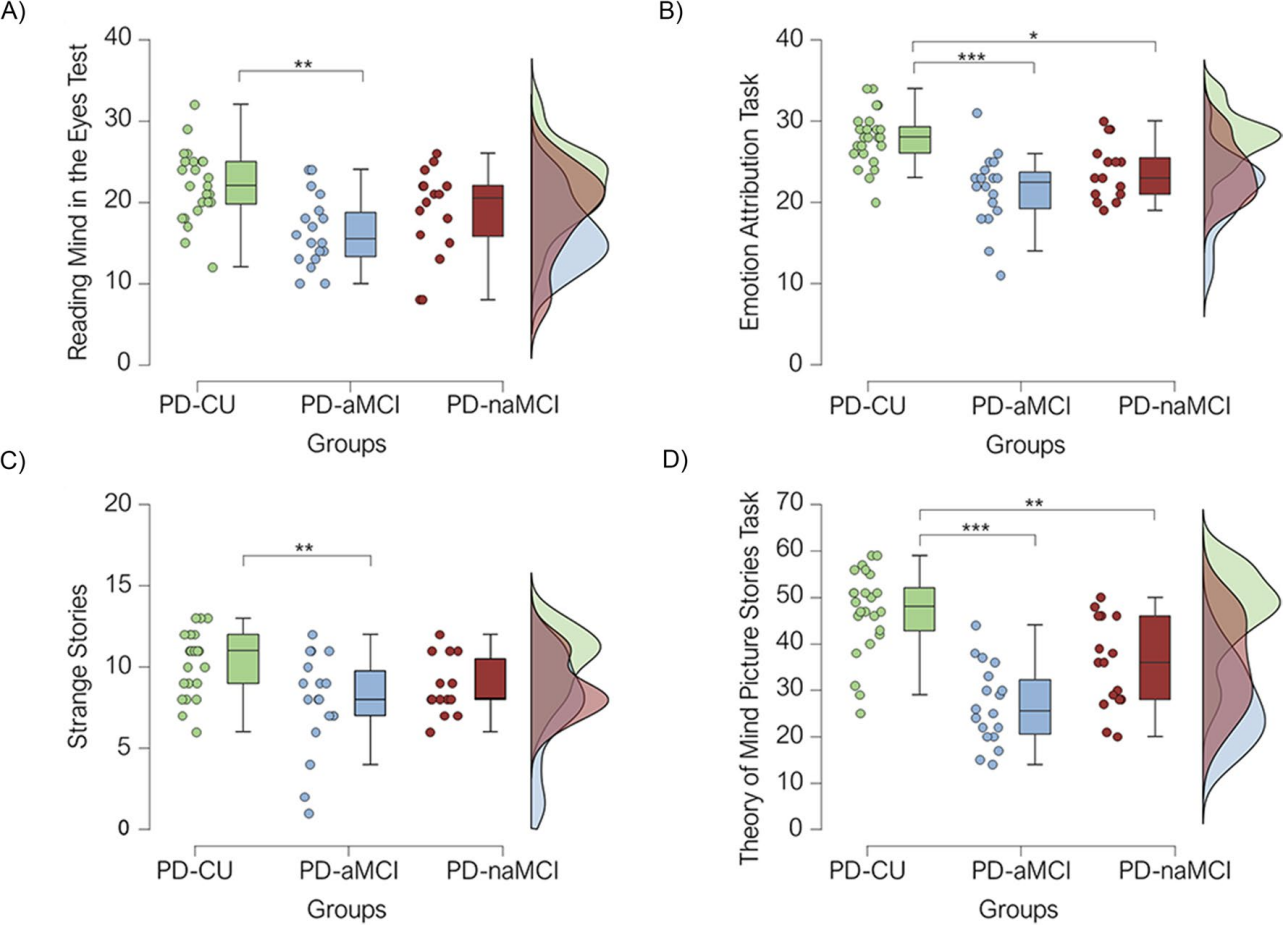
Raincloud plot representing performance in terms of accuracy. **A**) Reading Mind in the Eye Test; **B**) Emotion Attribution Task; **C**) Strange Stories; **D**) Theory of Mind Picture Stories Task across PD-CU, PD-aMCI, and PD-naMCI groups. Clouds represent the distribution, raindrops represent individual participants, and bars represent 95% confidence intervals. *p < 0.05; **p < 0.01; ***p < 0.001

Comparison between groups on SC tests showed that groups differed on RMET (emotion recognition/affective ToM) with PD-aMCI reporting poorer performance compared with PD-CU (*p* = 0.001), whereas no difference emerged between the PD-naMCI and PD-CU groups (*p* = 0.269; Fig. 3a). A general effect between groups also emerged on affective ToM (EAT), where both PD-aMCI and PD-naMCI scored lower than PD-CU (*p* < 0.001 and *p* = 0.012, respectively; Fig. 3b). Meanwhile, the scores of the three groups significantly differed on the Strange Stories task (Cognitive ToM), with PD-aMCI performing worse than PD-CU (*p* = 0.008), whereas no difference was found between PD-naMCI and PD-CU (*p* = 0.087; Fig. 3c).

At the same time, the three groups showed significant differences on the total score of the Theory of Mind Picture Stories Task (TMPS) (Cognitive ToM), where the PD-aMCI and PD-naMCI groups reported lower scores than PD-CU (*p* < 0.001 and *p* = 0.008, respectively; Fig. 3d). Moreover, we found group differences on first-order, second-order, and third-order false-belief comprehension questions; in particular, PD-aMCI scored worse than PD-CU on questions assessing first-order (*p* = 0.003) and third-order (*p* = 0.008) false belief, whereas both PD-aMCI and PD-naMCI groups reported lower performance on second-order false-belief question (Table 2). As for the TMPS subscores, we found significant differences on reality and reciprocity questions, where PD-aMCI scored worse than PD-CU, and on deception and cheating detection subscores, with PD-aMCI and PD-naMCI groups reporting poorer scores than PD-CU (Table 2).

**Table 2:** Comparison between PD groups on social cognition tests

	PD-CU (*n*=24) A	PD-aMCI (*n*=18) B	PD-naMCI (*n*=16) C		
	Mean ± SD	Mean **±** SD	Mean **±** SD	χ^2^	*p*
Emotion recognition/Affective ToM					
RMET	22.04 **±** 4.44	16.39 **±** 4.34	18.75 **±** 5.47	12.490	**0.002** **B<A**
EAT	27.87 **±** 3.34	21.56 **±** 4.54	23.87 **±** 3.52	20.731	**<0.001** **B, C<A**
Cognitive ToM					
ATT	10.33 **±** 1.95	7.78 **±** 3.04	8.79 **±** 1.8	10.152	**0.006** **B<A**
TMPST	46.92 **±** 9.15	26.78 **±** 8.48	35.5 **±** 9.74	27.998	**<0.001** **B, C<A**
First-order false beliefs	2.42 **±** 0.78	1.50 **±** 0.86	1.94 **±** 0.99	10.979	**0.004** **B<A**
Second-order false beliefs	2.46 **±** 0.98	0.94 **±** 0.99	1.63 **±** 0.88	19.135	**<0.001** **B, C<A**
Third-order false beliefs	2.17 **±** 0.96	1.28 **±** 0.89	1.56 **±** 0.81	9.853	**0.007** **B<A**
Reality	1.88 **±** 0.34	1.11 **±** 0.68	1.56 **±** 0.63	15.919	**<0.001** **B<A**
Reciprocity	2.83 **±** 0.38	2.17 **±** 0.79	2.63 **±** 0.5	10.378	**0.006** **B<A**
Deception	2.54 **±** 0.78	1.44 **±** 0.92	1.75 **±** 1.06	15.271	**<0.001** **B, C<A**
Cheating detection	1.96 **±** 0.36	1.44 **±** 0.7	1.44 **±** 0.63	10.662	**0.005** **B, C<A**

PD= Parkinson’s Disease; MCI= Mild Cognitive Impairment; PD-aMCI= PD patients with amnestic MCI; PD-naMCI= PD patients with non-amnestic MCI; SD= Standard Deviation; ToM= Theory of Mind; RMET= Reading the Mind in the Eyes Test; ATT= Advanced Test of ToM; EAT= Emotion Attribution Task; TMPS= Theory of Mind Picture Stories Task

Bold *p* values represent the statistically significant results

No relationship emerged between performance on social cognitive tests and clinical variables, including disease severity parameters, levodopa intake, and depression. Worse motor symptoms were related to poorer Executive control, whereas no effect of clinical variables on other cognitive domains was found.

## Discussion

In the present study, we examined the possible contribution of impairments in different cognitive domains, such as memory, language, and visuospatial functions, as mediators of the relationship between executive control and ToM (a component of social cognition) in PD. Memory impairments, together with language, mediated the relationship between executive control and poorer performance on ToM tests in PD. When exploring specific PD-MCI profiles and performance on social cognitive tests, impairments occurred more frequently in PD-aMCI patients than in PD-naMCI and PD controls. Finally, PD-aMCI patients scored worse on both emotion recognition/Affective ToM (i.e., EAT and RMET) and Cognitive ToM tasks (i.e., Strange Stories and TMPST) than PD controls, whereas the PD-naMCI group reported poorer performance only on EAT and TMPST.

### Mediation effects of cognitive domains in the relationship between executive control and social cognition in PD

We have previously shown a link between executive functions and ToM in PD (Maggi et al., 2022a, b), suggested as a consequence of frontostriatal changes after dopamine depletion in PD (Péron et al., 2009; Zgaljardic et al., 2006). To date, further studies have provided evidence of larger impaired circuitry due to dopamine depletion in PD (Rodriguez-Oroz et al., 2009), expanding deficits in associative components (Van Nuland et al., 2021), visuo-motor (Inzelberg et al., 2008), language (especially in spontaneous speech) (Liu et al., 2015; Tykalová et al., 2015), and memory (Banwinkler et al., 2022; Kulisevsky, 2000). In this study, we explored the idea that memory abilities affect social cognition, supported by mediation analyses. Indeed, memory performance mediated the relationship between executive control and EAT affective ToM performance, a test that requires participants to attribute how the characters of a story may feel in a particular situation. Hence, it is possible to hypothesize that the attribution of emotions to others is based on self-projection, modulated by familiarity with characters/situations, and possible past experiences (Moreau et al., 2015). In addition, performance on the TMPS (cognitive ToM) was mediated in PD by both language and memory abilities. Performance on this task involves the capacity to order events in a logical sequence, describe the overall story, and examine the comprehension of social features, such as deception, cheating, and reality knowledge. Considering the major impact of executive dysfunctions in ToM in PD, especially impaired cognitive flexibility (Maggi et al., 2022a, b), these results further confirm that impaired language alters the capacity to reason about mental states, comprehend questions, and understand false beliefs (Maggi et al., 2022a, b). Memory dysfunctions may contribute to difficulties in decoding social situations in PD, because the ability to recollect and reconstruct autobiographical past experiences might influence the imagination, attribution, and interaction with the intentions of others (Laurita & Spreng, 2017; Rubin et al., 2014). Finally, language abilities also contributed to the association between executive control and RMET (emotion recognition/affective ToM). This finding replicates previous evidence (Maggi et al., 2022a, b; Olderbak et al., 2015) whereby affective ToM processes relied on language abilities. Indeed, because optimal affective ToM requires semantic knowledge of emotional states as well as access to the mental lexicon to correctly choose the most appropriate label for the displayed emotions, altered language capacities in PD may predispose deficits in affective ToM. Overall, language and memory guide the maladaptive forms of social cognition observed in PD, supporting a complete overview of the underlying mechanisms subserving complex social operations.

### Social cognition in PD-MCI subtypes

Given the direct modulatory role of memory and language in ToM in PD, we aimed to expand its clinical relevance in a dedicated subsample analysis. Comparing patients with MCI with amnestic and non-amnestic characteristics, we observed a significantly higher prevalence of social cognitive impairment in amnestic MCI (14/18, 77.8%) than in PD-naMCI (3/16, 18.8%) and PD patients with preserved cognitive functions (2/24, 8.3%). In general MCI, failures in emotion recognition and ToM tasks have been reported (Bora & Yener, 2017). These results are in line with previous studies reporting a progressive impairment of social cognition due to the concomitant presence of PD-MCI (Alonso-Recio et al., 2021; Anderson et al., 2013), suggesting that PD-MCI patients are more susceptible to social cognitive impairment. If replicated, these findings suggest that PD pathophysiology alone is not responsible for frequent difficulties in social cognitive tasks. Indeed, social cognitive difficulties have also been found in PD patients with preserved cognitive functions, suggesting that social cognitive impairment is a major non-motor feature of PD, independent of cognitive status (Czernecki et al., 2021; Dodich et al., 2022).

More specifically, the prevalence rates of social cognitive impairment provided by these studies ranged from 30% (Dodich et al., 2022) to 64% (Czernecki et al., 2021) in PD-MCI patients and from 15% (Dodich et al., 2022) to 23% (Czernecki et al., 2021) in cognitively intact PD patients. By analyzing the same prevalence rates in our PD sample, we observed that 77.8% of PD-aMCI and 18.8% of PD-naMCI patients reported social cognitive impairment (50% considering both PD-MCI groups), whereas only two patients (8.3%) presented an isolated social cognitive impairment with preserved global cognitive functioning. To this, our rates are compatible with those presented in the aforementioned studies (Czernecki et al., 2021; Dodich et al., 2022), with a slight difference on the frequency of PD patients with social cognitive impairment alone. It is possible that these slight differences are explained by both the adoption of MDS level II criteria, which improves the accuracy of MCI diagnosis in PD compared to level I criteria (Czernecki et al., 2021), and the recruitment of a lower percentage (41%) of cognitively intact PD patients in our sample compared with previous studies (71% in (Dodich et al., 2022) and 85% in (Czernecki et al., 2021)). Hence, our findings suggest that MCI in PD seems to impact social cognition abilities not independently of global cognitive status as previously suggested (Alonso-Recio et al., 2021; Anderson et al., 2013). In general, the MCI non-PD population showed similar impairments in social cognitive tasks to the PD-aMCI population. Emotion recognition and ToM deficits have been observed in general MCI patients with predominant memory deficits (McCade et al., 2013; Michaelian et al., 2019, 2021). The neural basis explaining this finding is based on weaker functional connectivity between the frontal (i.e., dorsal medial prefrontal cortex, dorsolateral prefrontal cortex, and inferior frontal gyrus) and more posterior regions (i.e., temporal poles and the TPJ) (Moreau et al., 2015; Rabin et al., 2010; Spreng & Grady, 2010) in aMCI (Michaelian et al., 2021). These structures involved in the DMN are important in mentalizing networks (Abu-Akel, 2003; Schurz et al., 2014), because they are responsible for self-projection based on autobiographical memories, needed to engage others’ mental and affective states (Buckner and Carroll, 2007; Spreng et al., 2009). Individuals with aMCI also demonstrated volume reductions in key limbic structures, such as the hippocampus (Bharath et al., 2017; Michaelian et al., 2019). Hippocampal social functions are related to the navigation of social relationships (Kumaran et al., 2016; Tavares et al., 2015), perception of interpersonal cues (Rubin et al., 2014), and ToM operations, such as constructing and processing imagined scenes to represent others’ perspectives (Isaacowitz et al., 2007; Laurita & Spreng, 2017) and exploiting past experiences to infer or predict future outcomes (Bellmund et al., 2022; Reddy et al., 2021).

As part of PD pathophysiology, functional changes in the DMN (Chen et al., 2022), including key changes in PD along the DLPFC and vmPFC, have been linked to ToM dysfunctions (Díez-Cirarda et al., 2015; Ibarretxe-Bilbao et al., 2009; Kalbe et al., 2010b; Péron et al., 2009). Previous studies have shown that altered connectivity of the DMN is a pivotal neurophysiological mechanism of MCI in PD patients (Chen et al., 2022), especially in PD-aMCI (Rane et al., 2018; Ruppert et al., 2021). Moreover, volume reductions in structures involved in the limbic system, such as the hippocampus, have been reported in PD-MCI (Devignes et al., 2021). Altogether, our results provide the first evidence of social cognitive impairment associated with the amnestic profile of PD-MCI, probably explained by a common functional architecture from DMN changes (Michaelian et al., 2021) that possibly extends beyond hippocampal memory (Rabin et al., 2010; Spreng & Grady, 2010) and other frontostriatal structures that mediate cognitive downsides in social cognition.

#### Limitations

Some limitations of the present study should be addressed. First, no healthy controls were enrolled to compare performance on social cognitive tasks against PD subgroups. However, we explored social cognitive deficits in different subtypes of PD-MCI, because they represent a model to unravel the neuropathological frontostriatal mechanisms behind social cognitive deficits in humans. Hence, the presence of a cognitively preserved PD group was ideal for testing our predictions. Second, the limited number of patients within each PD-MCI subgroup could reduce the statistical power of the study, as well as the majority of multiple-domain MCI patients, which did not allow us to perform more sensitive analyses. However, power analysis revealed that our sample size was congruent with this analysis (see *Results* section), but studies including larger cohorts and exploring possible differences between single- and multiple-domain PD-MCI patients are needed to characterize how different PD-MCI profiles affect social cognitive abilities.

Moreover, the cross-sectional nature of the study did not allow us to infer the etiopathogenesis of social cognitive impairment in PD; future longitudinal studies may help to clarify the neurocognitive mechanisms underlying ToM deficits in PD and, more generally, neurodegenerative diseases. Finally, it should be noted that the tasks intervariability in the umbrella term “social cognition” together with the different stimuli and execution of these tasks do not allow uniquely to determine neurocognitive mechanisms behind social cognitive processes. Future studies should employ social cognitive tests that minimize the contribution of other cognitive domains, such as the Frith-Happe Animations task (White et al., 2011) to isolate some interrelated processes (e.g., nonverbal ToM processes). Nevertheless, these findings support the idea that the multidimensional nature of social cognition may enclose the contribution of different instrumental cognitive processes, such as memory, language, and visuospatial abilities.

## Conclusions

We demonstrated a novel contribution of memory and language dysfunctions, along with executive dysfunction, to social cognition, particularly ToM deficits in PD patients. Considering that PD-aMCI patients typically exhibit faster cognitive decline and a higher rate of conversion to dementia than those with PD-naMCI (Chung et al., 2019), early and subtle changes in social cognition may represent a behavioral marker of dementia. Hence, our findings suggest the need to routinely include social cognitive tests in clinical practice to identify patients presenting behaviors that disrupt the capacity to form and maintain interpersonal networks, determining withdrawal from social life (Desmarais et al., 2018; Eramudugolla et al., 2022). To this, timely identification of social cognitive impairment in patients has profound implications for therapeutic decision-making to avoid the risk of social isolation (Henry et al., 2016). Indeed, nonpharmacological interventions via role-play or social cognitive training comprising repeated practice of a range of social cognitive tasks have been associated with changes in the neural networks sustaining social cognitive processes (Hooker et al., 2013; Valk et al., 2017) and have proven to be effective in enhancing patients’ social skills and improving the quality of life of patients and caregivers (Kempnich et al., 2017; Kumfor et al., 2011).

### Supplementary Information

Below is the link to the electronic supplementary material.

Supplementary file1 (DOCX 17 KB)

Supplementary file2 (DOCX 25 KB)

Supplementary file3 (DOCX 26 KB)

Supplementary file4 (DOCX 26 KB)

Supplementary file5 (DOCX 27 KB)

## References

[CR1] Abu-Akel, A. (2003). A neurobiological mapping of theory of mind. *Brain Research Reviews**43*(1). 10.1016/S0165-0173(03)00190-5 10.1016/s0165-0173(03)00190-5 14499460

[CR2] Aiello, E. N., D’Iorio, A., Montemurro, S., Maggi, G., Giacobbe, C., Bari, V., ... Santangelo, G. (2022). Psychometrics, diagnostics and usability of Italian tools assessing behavioural and functional outcomes in neurological, geriatric and psychiatric disorders: a systematic review. *Neurological Sciences**43*(11). 10.1007/s10072-022-06300-8 10.1007/s10072-022-06300-8 PMC9616758 35932375

[CR3] Alonso-Recio L, Carvajal F, Merino C, Serrano JM (2021). Social Cognition and Cognitive Decline in Patients with Parkinson’s Disease. Journal of the International Neuropsychological Society.

[CR4] Anderson RJ, Simpson AC, Channon S, Samuel M, Brown RG (2013). Social problem solving, social cognition, and mild cognitive impairment in Parkinson’s disease. Behavioral Neuroscience.

[CR5] Banwinkler, M., Theis, H., Prange, S., & van Eimeren, T. (2022). Imaging the limbic system in Parkinson’s disease-a review of limbic pathology and clinical symptoms. *Brain Sciences*, *12*(9). 10.3390/brainsci12091248 10.3390/brainsci12091248 PMC9496800 36138984

[CR6] Baron-Cohen, S., Jolliffe, T., Mortimore, C., & Robertson, M. (1997). Another advanced test of theory of mind: Evidence from very high functioning adults with autism or Asperger syndrome. *Journal of Child Psychology and Psychiatry and Allied Disciplines*. 10.1111/j.1469-7610.1997.tb01599.x 10.1111/j.1469-7610.1997.tb01599.x 9363580

[CR7] Bellmund JLS, Deuker L, Montijn ND, Doeller CF (2022). Mnemonic construction and representation of temporal structure in the hippocampal formation. Nature Communications.

[CR8] Bharath, S., Joshi, H., John, J. P., Balachandar, R., Sadanand, S., Saini, J., ... Varghese, M. (2017). A multimodal structural and functional neuroimaging study of amnestic mild cognitive impairment. *American Journal of Geriatric Psychiatry*, *25*(2). 10.1016/j.jagp.2016.05.001 10.1016/j.jagp.2016.05.001 27555109

[CR9] Blair RJR, Cipolotti L (2000). Impaired social response reversal. A case of “acquired sociopathy”. Brain.

[CR10] Bodden, M. E., Dodel, R., & Kalbe, E. (2010). Theory of mind in Parkinson’s disease and related basal ganglia disorders: A systematic review. *Movement Disorders*. 10.1002/mds.22818 10.1002/mds.22818 19908307

[CR11] Bora, E., Walterfang, M., & Velakoulis, D. (2015). Theory of mind in Parkinson’s disease: A meta-analysis. *Behavioural**Brain Research**292*. 10.1016/j.bbr.2015.07.012 10.1016/j.bbr.2015.07.012 26166188

[CR12] Bora, E., & Yener, G. G. (2017). Meta-Analysis of social cognition in mild cognitive impairment. *Journal of Geriatric Psychiatry and Neurology*, *30*(4). 10.1177/0891988717710337 10.1177/0891988717710337 28639876

[CR13] Brüne, M. (2003). Theory of mind and the role of IQ in chronic disorganized schizophrenia. *Schizophrenia Research*. 10.1016/S0920-9964(02)00162-7 10.1016/s0920-9964(02)00162-7 12505138

[CR14] Buckner RL, Carroll DC (2007). Self-projection and the brain. Trends in cognitive sciences.

[CR15] Chen L, Huang T, Ma D, Chen Y-C (2022). Altered default mode network functional connectivity in Parkinson’s disease: A resting-state functional magnetic resonance imaging study. Frontiers in Neuroscience.

[CR16] Chenery, H. J., Angwin, A. J., & Copland, D. A. (2008). The basal ganglia circuits, dopamine, and ambiguous word processing: A neurobiological account of priming studies in Parkinson’s disease. *Journal of the International Neuropsychological Society**14*(3). 10.1017/S1355617708080491 10.1017/S1355617708080491 18419834

[CR17] Chung, S. J., Park, Y. H., Yun, H. J., Kwon, H., Yoo, H. S., Sohn, Y. H., ... Lee, P. H. (2019). Clinical relevance of amnestic versus non-amnestic mild cognitive impairment subtyping in Parkinson’s disease. *European Journal of Neurology*, *26*(5). 10.1111/ene.13886 10.1111/ene.13886 30565368

[CR18] Coricelli, G. (2005). Two-levels of mental states attribution: From automaticity to voluntariness. *Neuropsychologia*, *43*(2 SPEC. ISS.). 10.1016/j.neuropsychologia.2004.11.015 10.1016/j.neuropsychologia.2004.11.015 15707913

[CR19] Coundouris, S. P., Adams, A. G., Grainger, S. A., & Henry, J. D. (2019). Social perceptual function in parkinson’s disease: A meta-analysis. *Neuroscience and**Biobehavioral**Reviews**104*. 10.1016/j.neubiorev.2019.07.011 10.1016/j.neubiorev.2019.07.011 31336113

[CR20] Coundouris SP, Adams AG, Henry JD (2020). Empathy and theory of mind in Parkinson’s disease: A meta-analysis. Neuroscience and Biobehavioral Reviews.

[CR21] Czernecki V, Benchetrit E, Houot M, Pineau F, Mangone G, Corvol JC, Levy R (2021). Social cognitive impairment in early Parkinson’s disease: A novel “mild impairment”?. Parkinsonism and Related Disorders.

[CR22] Desmarais, P., Lanctôt, K. L., Masellis, M., Black, S. E., & Herrmann, N. (2018). Social inappropriateness in neurodegenerative disorders. *International**Psychogeriatrics**30*(2). 10.1017/S1041610217001260 10.1017/S1041610217001260 28689508

[CR23] Devignes, Q., Lopes, R., & Dujardin, K. (2022). Neuroimaging outcomes associated with mild cognitive impairment subtypes in Parkinson’s disease: A systematic review. *Parkinsonism and Related Disorders**95*. 10.1016/j.parkreldis.2022.02.006 10.1016/j.parkreldis.2022.02.006 35249807

[CR24] Devignes, Q., Viard, R., Betrouni, N., Carey, G., Kuchcinski, G., Defebvre, L., ... Dujardin, K. (2021). Posterior cortical cognitive deficits are associated with structural brain alterations in mild cognitive impairment in Parkinson’s disease. 10.3389/fnagi.2021.668559 10.3389/fnagi.2021.668559 PMC8155279 34054507

[CR25] Diamond A (2013). Executive functions. Annual Review of Psychology.

[CR26] Díez-Cirarda, M., Ojeda, N., Peña, J., Cabrera-Zubizarreta, A., Gómez-Beldarrain, M. Á., Gómez-Esteban, J. C., & Ibarretxe-Bilbao, N. (2015). Neuroanatomical correlates of theory of mind deficit in Parkinson’s disease: A multimodal imaging study. *PLoS ONE*, *10*(11). 10.1371/journal.pone.0142234 10.1371/journal.pone.0142234 PMC4641650 26559669

[CR27] Dodich, A., Funghi, G., Meli, C., Pennacchio, M., Longo, C., Malaguti, M. C., ... Papagno, C. (2022). Deficits in Emotion Recognition and Theory of Mind in Parkinson’s Disease Patients With and Without Cognitive Impairments. *Frontiers in Psychology,**13*, 866809. 10.3389/fpsyg.2022.866809 10.3389/fpsyg.2022.866809 PMC9138611 35645902

[CR28] Emre, M., Aarsland, D., Brown, R., Burn, D. J., Duyckaerts, C., Mizuno, Y., … Dubois, B. (2007). Clinical diagnostic criteria for dementia associated with Parkinson’s disease. *Movement Disorders**22*(12). 10.1002/mds.21507 10.1002/mds.21507 17542011

[CR29] Enrici, I., Mitkova, A., Castelli, L., Lanotte, M., Lopiano, L., & Adenzato, M. (2017). Deep Brain Stimulation of the subthalamic nucleus does not negatively affect social cognitive abilities of patients with Parkinson’s disease. *Scientific Reports*, *7*(1). 10.1038/s41598-017-09737-6 10.1038/s41598-017-09737-6 PMC5573348 28842656

[CR30] Enticott, P. G., & Ogloff, J. R. P. (2006). Elucidation of impulsivity. *Australian Psychologist*, *41*(1). 10.1080/00050060500391894

[CR31] Eramudugolla, R., Huynh, K., Zhou, S., Amos, J. G., & Anstey, K. J. (2022). Social cognition and social functioning in MCI and Dementia in an epidemiological sample. *Journal of the International Neuropsychological Society*, *28*(7). 10.1017/S1355617721000898 10.1017/S1355617721000898 34486512

[CR32] Faul, F., Erdfelder, E., Lang, A. G., & Buchner, A. (2007). G*Power 3: A flexible statistical power analysis program for the social, behavioral, and biomedical sciences. *Behavior Research Methods*, *39*(2). 10.3758/BF03193146 10.3758/bf03193146 17695343

[CR33] Fitch, W. T., Huber, L., & Bugnyar, T. (2010). Social cognition and the evolution of language: Constructing cognitive phylogenies. *Neuron**65*(6). 10.1016/j.neuron.2010.03.011 10.1016/j.neuron.2010.03.011 PMC4415479 20346756

[CR34] Foley, J. A., Lancaster, C., Poznyak, E., Borejko, O., Niven, E., Foltynie, T., ... Cipolotti, L. (2019). Impairment in theory of mind in Parkinson’s disease is explained by deficits in inhibition. *Parkinson’s Disease*, *2019*. 10.1155/2019/5480913 10.1155/2019/5480913 PMC6558602 31275544

[CR35] Friedman, N. P., & Miyake, A. (2004). The relations among inhibition and interference control functions: A Latent-variable analysis. *Journal of Experimental Psychology: General*, *133*(1). 10.1037/0096-3445.133.1.101 10.1037/0096-3445.133.1.101 14979754

[CR36] Goldman, J. G., Holden, S., Ouyang, B., Bernard, B., Goetz, C. G., & Stebbins, G. T. (2015). Diagnosing PD-MCI by MDS task force criteria: How many and which neuropsychological tests? *Movement Disorders*, *30*(3). 10.1002/mds.26084 10.1002/mds.26084 PMC4357536 25449653

[CR37] Happé, F. G. E. (1994). An advanced test of theory of mind: Understanding of story characters’ thoughts and feelings by able autistic, mentally handicapped, and normal children and adults. *Journal of Autism and Developmental Disorders*. 10.1007/BF02172093 10.1007/BF02172093 8040158

[CR38] Henry, J. D., Von Hippel, W., Molenberghs, P., Lee, T., & Sachdev, P. S. (2016). Clinical assessment of social cognitive function in neurological disorders. *Nature Reviews Neurology**12*(1). 10.1038/nrneurol.2015.229 10.1038/nrneurol.2015.229 26670297

[CR39] Hooker, C. I., Bruce, L., Fisher, M., Verosky, S. C., Miyakawa, A., D’Esposito, M., & Vinogradov, S. (2013). The influence of combined cognitive plus social-cognitive training on amygdala response during face emotion recognition in schizophrenia. *Psychiatry Research - Neuroimaging*, *213*(2). 10.1016/j.pscychresns.2013.04.001 10.1016/j.pscychresns.2013.04.001 PMC6999046 23746615

[CR40] Hughes, A. J., Daniel, S. E., Kilford, L., & Lees, A. J. (1992). Accuracy of clinical diagnosis of idiopathic Parkinson’s disease: A clinico-pathological study of 100 cases. *Journal of Neurology Neurosurgery and Psychiatry*, *55*(3). 10.1136/jnnp.55.3.181 10.1136/jnnp.55.3.181 PMC1014720 1564476

[CR41] Ibarretxe-Bilbao, N., Junque, C., Tolosa, E., Marti, M. J., Valldeoriola, F., Bargallo, N., & Zarei, M. (2009). Neuroanatomical correlates of impaired decision-making and facial emotion recognition in early Parkinson’s disease. *European Journal of Neuroscience*, *30*(6). 10.1111/j.1460-9568.2009.06892.x 10.1111/j.1460-9568.2009.06892.x 19735293

[CR42] IBM. (2019). IBM SPSS statistics for windows (Version 26.0) Armonk, NY: IBM Corp.

[CR43] Inzelberg, R., Schechtman, E., & Hocherman, S. (2008). Visuo-motor coordination deficits and motor impairments in Parkinson’s disease. *PLoS**ONE*, *3*(11). 10.1371/journal.pone.0003663 10.1371/journal.pone.0003663 PMC2576439 18987752

[CR44] Isaacowitz, D. M., Löckenhoff, C. E., Lane, R. D., Wright, R., Sechrest, L., Riedel, R., & Costa, P. T. (2007). Age differences in recognition of emotion in lexical stimuli and facial expressions. *Psychology and Aging*, *22*(1). 10.1037/0882-7974.22.1.147 10.1037/0882-7974.22.1.147 17385991

[CR45] Kalbe, E., Schlegel, M., Sack, A. T., Nowak, D. A., Dafotakis, M., Bangard, C., ... Kessler, J. (2010a). Dissociating cognitive from affective theory of mind: A TMS study. *Cortex*. 10.1016/j.cortex.2009.07.010 10.1016/j.cortex.2009.07.010 19709653

[CR46] Kalbe, E., Schlegel, M., Sack, A. T., Nowak, D. A., Dafotakis, M., Bangard, C., ... Kessler, J. (2010). Dissociating cognitive from affective theory of mind: A TMS study. *Cortex,**46*(6), 769–780. 10.1016/j.cortex.2009.07.010 10.1016/j.cortex.2009.07.010 19709653

[CR47] Kempnich, C. L., Wong, D., Georgiou-Karistianis, N., & Stout, J. C. (2017). Feasibility and Efficacy of Brief Computerized Training to Improve Emotion Recognition in Premanifest and Early-Symptomatic Huntington’s Disease. *Journal of the International Neuropsychological Society*, *23*(4). 10.1017/S1355617717000145 10.1017/S1355617717000145 28357975

[CR48] Kosutzka Z, Kralova M, Kusnirova A, Papayova M, Valkovic P, Csefalvay Z, Hajduk M (2019). Neurocognitive Predictors of Understanding of Intentions in Parkinson Disease. Journal of Geriatric Psychiatry and Neurology.

[CR49] Kulisevsky, J. (2000). Role of dopamine in learning and memory: Implications for the treatment of cognitive dysfunction in patients with Parkinson’s disease. *Drugs and Aging**16*(5). 10.2165/00002512-200016050-00006 10.2165/00002512-200016050-00006 10917074

[CR50] Kumaran, D., Banino, A., Blundell, C., Hassabis, D., & Dayan, P. (2016). Computations underlying social hierarchy learning: Distinct neural mechanisms for updating and representing self-relevant information. *Neuron*, *92*(5). 10.1016/j.neuron.2016.10.052 10.1016/j.neuron.2016.10.052 PMC5158095 27930904

[CR51] Kumfor, F., Miller, L., Lah, S., Hsieh, S., Savage, S., Hodges, J. R., & Piguet, O. (2011). Are you really angry? The effect of intensity on facial emotion recognition in frontotemporal dementia. *Social Neuroscience*, *6*(5–6). 10.1080/17470919.2011.620779 10.1080/17470919.2011.620779 21957889

[CR52] Laillier, R., Viard, A., Caillaud, M., Duclos, H., Bejanin, A., de La Sayette, V., ... Laisney, M. (2019). Neurocognitive determinants of theory of mind across the adult lifespan. *Brain and Cognition*, *136*. 10.1016/j.bandc.2019.103588 10.1016/j.bandc.2019.103588 31419764

[CR53] Laurita, A. C., & Spreng, R. N. (2017). The hippocampus and social cognition. *The Hippocampus from Cells to Systems: Structure, Connectivity, and Functional Contributions to Memory and Flexible Cognition*. 10.1007/978-3-319-50406-3_17

[CR54] Litvan, I., Goldman, J. G., Tröster, A. I., Schmand, B. A., Weintraub, D., Petersen, R. C., ... Emre, M. (2012). Diagnostic criteria for mild cognitive impairment in Parkinson’s disease: Movement Disorder Society Task Force guidelines. *Movement Disorders,**27*(3), 349–356. 10.1002/mds.24893 10.1002/mds.24893 PMC3641655 22275317

[CR55] Liu, L., Luo, X. G., Dy, C. L., Ren, Y., Feng, Y., Yu, H. M., ... He, Z. Y. (2015). Characteristics of language impairment in Parkinson’s disease and its influencing factors. *Translational Neurodegeneration*, *4*(1). 10.1186/2047-9158-4-2 10.1186/2047-9158-4-2 PMC4328233 25685335

[CR56] MacOir, J., Fossard, M., Mérette, C., Langlois, M., Chantal, S., & Auclair-Ouellet, N. (2013). The role of basal ganglia in language production: Evidence from Parkinson’s disease. *Journal of**Parkinson’s Disease*, *3*(3). 10.3233/JPD-130182 10.3233/JPD-130182 23948988

[CR57] Maggi, G., Cima Muñoz, A. M., Obeso, I., & Santangelo, G. (2022). Neuropsychological, neuropsychiatric, and clinical correlates of affective and cognitive theory of mind in Parkinson’s disease: A meta-analysis. *Neuropsychology*. 10.1037/neu0000807 10.1037/neu0000807 35389722

[CR58] Maggi G, Di Meglio D, Vitale C, Amboni M, Obeso I, Santangelo G (2022). The impact of executive dysfunctions on Theory of Mind abilities in Parkinson’s disease. Neuropsychologia.

[CR59] Maggi, G., D’Iorio, A., Aiello, E. N., Poletti, B., Ticozzi, N., Silani, V., ... Santangelo, G. (2023). Psychometrics and diagnostics of the Italian version of the Beck Depression Inventory-II (BDI-II) in Parkinson’s disease. *Neurological Sciences : Official Journal of the Italian Neurological Society and of**the Italian Society of Clinical Neurophysiology*. 10.1007/s10072-023-06619-w 10.1007/s10072-023-06619-w PMC10102079 36653542

[CR60] McCade, D., Savage, G., Guastella, A., Lewis, S. J. G., & Naismith, S. L. (2013). Emotion recognition deficits exist in mild cognitive impairment, but only in the amnestic subtype. *Psychology and Aging*, *28*(3). 10.1037/a0033077 10.1037/a0033077 24041005

[CR61] McKinlay, A., Albicini, M., & Kavanagh, P. S. (2013). The effect of cognitive status and visuospatial performance on affective theory of mind in Parkinson’s disease. *Neuropsychiatric Disease and Treatment*, *9*. 10.2147/NDT.S49104 10.2147/NDT.S49104 PMC3760454 24019747

[CR62] Michaelian, J. C., Duffy, S. L., Mowszowski, L., Guastella, A. J., McCade, D., McKinnon, A. C., & Naismith, S. L. (2021). Poorer Theory of Mind in Amnestic Mild Cognitive Impairment Is Associated with Decreased Functional Connectivity in the Default Mode Network. *Journal of**Alzheimer’s Disease*, *81*(3). 10.3233/JAD-201284 10.3233/JAD-201284 33843670

[CR63] Michaelian JC, Mowszowski L, Guastella AJ, Henry JD, Duffy S, McCade D, Naismith SL (2019). Theory of mind in mild cognitive impairment - Relationship with limbic structures and behavioural change. Journal of the International Neuropsychological Society.

[CR64] Moreau N, Rauzy S, Bonnefoi B, Renié L, Martinez-Almoyna L, Viallet F, Champagne-Lavau M (2015). Different Patterns of Theory of Mind Impairment in Mild Cognitive Impairment. Journal of Alzheimer’s Disease.

[CR65] Oakley, B. F. M., Brewer, R., Bird, G., & Catmur, C. (2016). Theory of mind is not theory of emotion: A cautionary note on the reading the mind in the eyes test. *Journal of Abnormal Psychology*. 10.1037/abn0000182 10.1037/abn0000182 PMC4976760 27505409

[CR66] Obeso, I., Wilkinson, L., Casabona, E., Bringas, M. L., Álvarez, M., Álvarez, L., ... Jahanshahi, M. (2011). Deficits in inhibitory control and conflict resolution on cognitive and motor tasks in Parkinson’s disease. *Experimental Brain Research,**212*(3), 371–384. 10.1007/s00221-011-2736-6 10.1007/s00221-011-2736-6 21643718

[CR67] Olderbak, S., Wilhelm, O., Olaru, G., Geiger, M., Brenneman, M. W., & Roberts, R. D. (2015). A psychometric analysis of the reading the mind in the eyes test: Toward a brief form for research and applied settings. *Frontiers in Psychology*, *6*(OCT). 10.3389/fpsyg.2015.01503 10.3389/fpsyg.2015.01503 PMC4593947 26500578

[CR68] Olson, I. R., McCoy, D., Klobusicky, E., & Ross, L. A. (2013). Social cognition and the anterior temporal lobes: A review and theoretical framework. *Social Cognitive and Affective Neuroscience*, *8*(2). 10.1093/scan/nss119 10.1093/scan/nss119 PMC3575728 23051902

[CR69] Péron, J., le Jeune, F., Haegelen, C., Dondaine, T., Drapier, D., Sauleau, P., ... Vérin, M. (2010). Subthalamic nucleus stimulation affects theory of mind network: A PET study in Parkinson’s disease. *PLoS ONE*. 10.1371/journal.pone.0009919 10.1371/journal.pone.0009919 PMC2847915 20360963

[CR70] Péron, J., Vicente, S., Leray, E., Drapier, S., Drapier, D., Cohen, R., ... Vérin, M. (2009). Are dopaminergic pathways involved in theory of mind? A study in Parkinson’s disease. *Neuropsychologia,**47*(2), 406–414. 10.1016/j.neuropsychologia.2008.09.008 10.1016/j.neuropsychologia.2008.09.008 18845171

[CR71] Phillips, A. T., Wellman, H. M., & Spelke, E. S. (2002). Infants’ ability to connect gaze and emotional expression to intentional action. *Cognition*, *85*(1). 10.1016/S0010-0277(02)00073-2 10.1016/s0010-0277(02)00073-2 12086713

[CR72] Pineda-Pardo, J. D. S. A., Sánchez-Ferro, Á., Monje, M. H. G., Pavese, N., & Obeso, J. A. (2022). Onset pattern of nigrostriatal denervation in early Parkinson’s disease. *Brain*, *145*(3). 10.1093/brain/awab378 10.1093/brain/awab378 PMC9351472 35349639

[CR73] Preacher KJ, Hayes AF (2008). Asymptotic and resampling strategies for assessing and comparing indirect effects in multiple mediator models. Behavior Research Methods.

[CR74] Quesque, F., & Rossetti, Y. (2020). What Do Theory-of-Mind Tasks Actually Measure? Theory and Practice. *Perspectives on Psychological Science*, *15*(2). 10.1177/1745691619896607 10.1177/1745691619896607 32069168

[CR75] Rabin, J. S., Gilboa, A., Stuss, D. T., Mar, R. A., & Rosenbaum, R. S. (2010). Common and unique neural correlates of autobiographical memory and theory of mind. *Journal of Cognitive Neuroscience*, *22*(6). 10.1162/jocn.2009.21344 10.1162/jocn.2009.21344 19803685

[CR76] Rane, S., Donahue, M. J., & Claassen, D. O. (2018). Amnestic mild cognitive impairment individuals with dissimilar pathologic origins show common regional vulnerability in the default mode network. *Alzheimer’s and Dementia: Diagnosis, Assessment and Disease Monitoring*, *10*. 10.1016/j.dadm.2018.08.004 10.1016/j.dadm.2018.08.004 PMC6258224 30511009

[CR77] Reddy, L., Zoefel, B., Possel, J. K., Peters, J., Dijksterhuis, D. E., Poncet, M., ... Self, M. W. (2021). Human hippocampal neurons track moments in a sequence of events. *Journal of Neuroscience*, *41*(31). 10.1523/JNEUROSCI.3157-20.2021 10.1523/JNEUROSCI.3157-20.2021 PMC8336696 34183446

[CR78] Rodriguez-Oroz, M. C., Jahanshahi, M., Krack, P., Litvan, I., Macias, R., Bezard, E., & Obeso, J. A. (2009). Initial clinical manifestations of Parkinson’s disease: features and pathophysiological mechanisms. *The Lancet Neurology**8*(12). 10.1016/S1474-4422(09)70293-5 10.1016/S1474-4422(09)70293-5 19909911

[CR79] Rubin, R. D., Watson, P. D., Duff, M. C., & Cohen, N. J. (2014). The role of the hippocampus in flexible cognition and social behavior. *Frontiers in Human Neuroscience**8*(SEP). 10.3389/fnhum.2014.00742 10.3389/fnhum.2014.00742 PMC4179699 25324753

[CR80] Ruppert, M. C., Greuel, A., Freigang, J., Tahmasian, M., Maier, F., Hammes, J., ... Eggers, C. (2021). The default mode network and cognition in Parkinson’s disease: A multimodal resting-state network approach. *Human Brain Mapping*, *42*(8). 10.1002/hbm.25393 10.1002/hbm.25393 PMC8090788 33638213

[CR81] Sagliano, L., Maiese, B., & Trojano, L. (2020). Danger is in the eyes of the beholder: The effect of visible and invisible affective faces on the judgment of social interactions. *Cognition*, *203*. 10.1016/j.cognition.2020.104371 10.1016/j.cognition.2020.104371 32569893

[CR82] Saltzman J, Strauss E, Hunter M, Archibald S (2000). Theory of mind and executive functions in normal human aging and Parkinson’s disease. Journal of the International Neuropsychological Society.

[CR83] Santangelo, G., Siciliano, M., Pedone, R., Vitale, C., Falco, F., Bisogno, R., ... Trojano, L. (2015). Normative data for the Montreal Cognitive Assessment in an Italian population sample. *Neurological Sciences*. 10.1007/s10072-014-1995-y 10.1007/s10072-014-1995-y 25380622

[CR84] Sawada, Y., Nishio, Y., Suzuki, K., Hirayama, K., Takeda, A., Hosokai, Y., ... Mori, E. (2012). Attentional set-shifting deficit in parkinson’s disease is associated with prefrontal dysfunction: An FDG-PET study. *PLoS**ONE*, *7*(6). 10.1371/journal.pone.0038498 10.1371/journal.pone.0038498 PMC3369918 22685575

[CR85] Schurz M, Radua J, Aichhorn M, Richlan F, Perner J (2014). Fractionating theory of mind: A meta-analysis of functional brain imaging studies. Neuroscience and Biobehavioral Reviews.

[CR86] Shamay-Tsoory SG, Aharon-Peretz J (2007). Dissociable prefrontal networks for cognitive and affective theory of mind: A lesion study. Neuropsychologia.

[CR87] Shamay-Tsoory, S. G., Tomer, R., Goldsher, D., Berger, B. D., & Aharon-Peretz, J. (2004). Impairment in cognitive and affective empathy in patients with brain lesions: Anatomical and cognitive correlates. *Journal of Clinical and Experimental Neuropsychology*. 10.1080/13803390490515531 10.1080/13803390490515531 15590464

[CR88] Spreng RN, Mar RA, Kim AS (2009). The common neural basis of autobiographical memory, prospection, navigation, theory of mind, and the default mode: a quantitative meta-analysis. Journal of cognitive neuroscience.

[CR89] Spreng, R. N., & Grady, C. L. (2010). Patterns of brain activity supporting autobiographical memory, prospection, and theory of mind, and their relationship to the default mode network. *Journal of Cognitive Neuroscience*, *22*(6). 10.1162/jocn.2009.21282 10.1162/jocn.2009.21282 19580387

[CR90] Tavares, R. M., Mendelsohn, A., Grossman, Y., Williams, C. H., Shapiro, M., Trope, Y., & Schiller, D. (2015). A map for social navigation in the human brain. *Neuron*, *87*(1). 10.1016/j.neuron.2015.06.011 10.1016/j.neuron.2015.06.011 PMC4662863 26139376

[CR91] Tykalová, T., Rusz, J., Čmejla, R., Klempíř, J., Růžičková, H., Roth, J., & Růžička, E. (2015). Effect of dopaminergic medication on speech dysfluency in Parkinson’s disease: a longitudinal study. *Journal of Neural Transmission*, *122*(8). 10.1007/s00702-015-1363-y 10.1007/s00702-015-1363-y 25583417

[CR92] Valk, S. L., Bernhardt, B. C., Trautwein, F. M., Böckler, A., Kanske, P., Guizard, N., ... Singer, T. (2017). Structural plasticity of the social brain: Differential change after socio-affective and cognitive mental training. *Science Advances*, *3*(10). 10.1126/sciadv.1700489 10.1126/sciadv.1700489 PMC5627980 28983507

[CR93] Van Nuland, A. J., Helmich, R. C., Dirkx, M. F., Zach, H., Toni, I., Cools, R., & Den Ouden, H. E. M. (2021). Effects of dopamine on reinforcement learning in Parkinson’s disease depend on motor phenotype. *Brain*, *143*(11). 10.1093/BRAIN/AWAA335 10.1093/brain/awaa335 PMC7719026 33147621

[CR94] White, S. J., Coniston, D., Rogers, R., & Frith, U. (2011). Developing the Frith-Happé animations: A quick and objective test of Theory of Mind for adults with autism. *Autism Research*, *4*(2). 10.1002/aur.174 10.1002/aur.174 21480540

[CR95] Zgaljardic D, Borod J, Foldi N, Mattis P, Gordon M, Feigin A, Eidelberg D (2006). An examination of executive dysfunction associated with frontostriatal circuitry in Parkinson’s disease. Journal of Clinical and Experimental Neuropsychology.

