# Supplementary material for: Theory of mind in mild cognitive impairment and Parkinson’s disease: The role of memory impairment
Source: Cogn Affect Behav Neurosci. 2023 Dec 4;24(1):156–70. doi: 10.3758/s13415-023-01142-z (PMC10827829; doi:10.3758/s13415-023-01142-z)
Supplement: Supplementary file 1 — Supplementary file1 (DOCX 17 KB) [file 13415_2023_1142_MOESM1_ESM.docx]

**Supplementary material – References of tools used in the paper**

*- Montreal Cognitive Assessment*

Z.S. Nasreddine, N.A. Phillips, V. Bedirian, S. Charbonneau, V. Whitehead, I. Collin, H. Chertkow, The Montreal cognitive assessment, MoCA: A brief screening tool for mild cognitive impairment, J. Am. Geriatr. Soc. 53 (2005) 695-699.

G. Santangelo, M. Siciliano, R. Pedone, C. Vitale, F. Falco, R. Bisogno, P. Siano, P. Barone, D. Grossi, F. Santangelo, L. Trojano, Normative data for the Montreal Cognitive Assessment in an Italian population sample, Neurol. Sci. 36 (2015) 585-591.

*- Dimensional Apathy Scale*

R. Radakovic, S. Abrahams, Developing a new apathy measurement scale: Dimensional Apathy Scale, Psychiatry Res. 219 (2014) 658-663.

G. Santangelo, S. Raimo, M. Siciliano, A. D'Iorio, F. Piscopo, S. Cuoco, M. Bottone, F. Trojsi, D. Grossi, L. Trojano, Assessment of apathy independent of physical disability: validation of the Dimensional Apathy Scale in Italian healthy sample, Neurol. Sci. 38 (2017) 303-309.

G. Santangelo, A. D’Iorio, F. Piscopo, S. Cuoco, K. Longo, M. Amboni, C. Baiano, D. Tafuri. M. T. Pellecchia, P. Barone, C. Vitale, Assessment of apathy minimising the effect of motor dysfunctions in Parkinson’s Disease: a validation study of the Dimensional Apathy Scale, Qual. Life Res. 26 (2017) 2533-2540.

*- Rey Auditory Verbal Learning Test*

G. A. Carlesimo, C. Caltagirone, G. Gainotti, The Mental Deterioration Battery: normative data, diagnostic reliability and qualitative analyses of cognitive impairment, Eur. Neurol. 36 (1996) 378–384.

*- Trail Making Test*

A. R. Giovagnoli, M. Del Pesce, S. Mascheroni, M. Simoncelli, M. Laiacona, E. Capitani, Trail making test: norma-tive values from 287 normal adult controls, Ital. J. Neurol. Sci. 17 (1996) 305–309.

*- Stroop Test*

Barbarotto, M. Laiacona, R. Frosio, M. Vecchio, A. Farinato, E. Capitani, A normative study on visual reaction times and two Stroop colour word tests, Ital. J. Neurol. Sci. 19 (1998) 161–170.

- *Benton Judgment of Line Orientation Test*

A. L. Benton, N. R. Varney, K. D. Hamsher, Visuospatial judgment: a clinical test, Arch. Neurol. 35 (1978) 364– 367.

- *Constructional Apraxia Test*

Grossi, D., & Trojano, L, Constructional apraxia. In G. Denes & L. Pizzamiglio (Eds.), *Handbook of clinical and experimental neuropsychology* (pp. 441–450) (1999). Psychology Press/Erlbaum (UK) Taylor & Francis.

-*Short story of Anna Pesenti*

Novelli, G., et al. "Tre test clinici di memoria verbale a lungo termine: taratura su soggetti normali." *Archivio di psicologia, neurologia e psichiatria* (1986).

-*Esame Neuropsicologico per l’Afasia*

Capasso, R., & Miceli, G. (2001). *Esame Neuropsicologico per l'Afasia: ENPA* (Vol. 4). Springer Science & Business Media.
