# Supplementary material for: Theory of mind in mild cognitive impairment and Parkinson’s disease: The role of memory impairment
Source: Cogn Affect Behav Neurosci. 2023 Dec 4;24(1):156–70. doi: 10.3758/s13415-023-01142-z (PMC10827829; doi:10.3758/s13415-023-01142-z)
Supplement: Supplementary file 2 — Supplementary file2 (DOCX 25 KB) [file 13415_2023_1142_MOESM2_ESM.docx]

**Supplementary Material 2.**

| ***Predictor*** | | | | | | | |
| --- | --- | --- | --- | --- | --- | --- | --- |
| Color Reading – Stroop Test | | | | | | | |
| *Direct Effect* | | | | | | | |
| **95% Confidence Interval** | | | | | | | |
|  |  | *Estimate* | *SE* | *t* | *p* | *Lower* | *Upper* |
| Color Reading -> RMET | | 0.151 | 0.053 | 2.816 | **0.007** | **0.043** | **0.258** |
| *Indirect Effects* | | | | | | | |
| **95% Confidence Interval** | | | | | | | |
|  |  | *Estimate* | | *BootSE* | | *BootLower* | *BootUpper* |
| Total |  | 0.071 | | 0.039 | | **0.002** | **0.156** |
| Color Reading -> Language -> RMET | | 0.033 | | 0.027 | | -0.012 | 0.092 |
| Color Reading -> Memory -> RMET | | 0.031 | | 0.025 | | -0.006 | 0.091 |
| Color Reading -> Visuospatial -> RMET | | 0.007 | | 0.025 | | -0.040 | 0.063 |
| *Total Effect* |  |  |  |  | |  |  |
| **95% Confidence Interval** | | | | | | | |
|  |  | *Estimate* | *SE* | *t* | *p* | *Lower* | *Upper* |
| Color Reading -> RMET | | 0.222 | 0.052 | 4.248 | **<0.001** | **0.117** | **0.327** |

Mediation Models with Reading Mind in the Eye Test (RMET) as outcome and single executive functions test as independent variable.

| ***Predictor*** | | | | | | | |
| --- | --- | --- | --- | --- | --- | --- | --- |
| Interference task – Stroop Test | | | | | | | |
| *Direct Effect* | | | | | | | |
| **95% Confidence Interval** | | | | | | | |
|  |  | *Estimate* | *SE* | *t* | *p* | *Lower* | *Upper* |
| Interference task -> RMET | | 0.223 | 0.082 | 2.726 | **0.009** | **0.059** | **0.387** |
| *Indirect Effects* | | | | | | | |
| **95% Confidence Interval** | | | | | | | |
|  |  | *Estimate* | | *BootSE* | | *BootLower* | *BootUpper* |
| Total |  | 0.115 | | 0.057 | | **0.016** | **0.236** |
| Interference task -> Language -> RMET | | 0.062 | | 0.036 | | **0.001** | **0.140** |
| Interference task -> Memory -> RMET | | 0.050 | | 0.036 | | -0.003 | 0.136 |
| Interference task -> Visuospatial -> RMET | | 0.003 | | 0.044 | | -0.087 | 0.094 |
| *Total Effect* |  |  |  |  | |  |  |
| **95% Confidence Interval** | | | | | | | |
|  |  | *Estimate* | *SE* | *t* | *p* | *Lower* | *Upper* |
| Interference task -> RMET | | 0.338 | 0.075 | 4.491 | **<0.001** | **0.187** | **0.489** |

| ***Predictor*** | | | | | | | |
| --- | --- | --- | --- | --- | --- | --- | --- |
| Trail Making Test part A (TMT:A) | | | | | | | |
| *Direct Effect* | | | | | | | |
| **95% Confidence Interval** | | | | | | | |
|  |  | *Estimate* | *SE* | *t* | *p* | *Lower* | *Upper* |
| TMT:A -> RMET | | -0.035 | 0.013 | -2.761 | **0.008** | **-0.061** | **-0.010** |
| *Indirect Effects* | | | | | | | |
| **95% Confidence Interval** | | | | | | | |
|  |  | *Estimate* | | *BootSE* | | *BootLower* | *BootUpper* |
| Total |  | -0.017 | | 0.011 | | -0.043 | 0.001 |
| TMT:A -> Language -> RMET | | -0.010 | | 0.008 | | **-0.032** | **-0.001** |
| TMT:A -> Memory -> RMET | | -0.008 | | 0.005 | | **-0.019** | **-0.001** |
| TMT:A -> Visuospatial -> RMET | | 0.001 | | 0.008 | | -0.014 | 0.017 |
| *Total Effect* |  |  |  |  | |  |  |
| **95% Confidence Interval** | | | | | | | |
|  |  | *Estimate* | *SE* | *t* | *p* | *Lower* | *Upper* |
| TMT:A -> RMET | | -0.053 | 0.012 | -4.412 | **0.001** | **-0.076** | **-0.029** |

| ***Predictor*** | | | | | | | |
| --- | --- | --- | --- | --- | --- | --- | --- |
| Trail Making Test B-A (TMT:B-A) | | | | | | | |
| *Direct Effect* | | | | | | | |
| **95% Confidence Interval** | | | | | | | |
|  |  | *Estimate* | *SE* | *t* | *p* | *Lower* | *Upper* |
| TMT:B-A -> RMET | | -0.012 | 0.006 | -2.237 | **0.030** | **-0.024** | **-0.001** |
| *Indirect Effects* | | | | | | | |
| **95% Confidence Interval** | | | | | | | |
|  |  | *Estimate* | | *BootSE* | | *BootLower* | *BootUpper* |
| Total |  | -0.007 | | 0.004 | | **-0.015** | **-0.001** |
| TMT:B-A -> Language -> RMET | | -0.004 | | 0.003 | | -0.010 | 0.001 |
| TMT:B-A -> Memory -> RMET | | -0.003 | | 0.002 | | -0.008 | 0.001 |
| TMT:B-A -> Visuospatial -> RMET | | -0.001 | | 0.003 | | -0.006 | 0.005 |
| *Total Effect* |  |  |  |  | |  |  |
| **95% Confidence Interval** | | | | | | | |
|  |  | *Estimate* | *SE* | *t* | *p* | *Lower* | *Upper* |
| TMT:B-A -> RMET | | -0.019 | 0.005 | -3.595 | **0.001** | **-0.030** | **-0.009** |

| ***Predictor*** | | | | | | | |
| --- | --- | --- | --- | --- | --- | --- | --- |
| Phonological Fluency | | | | | | | |
| *Direct Effect* | | | | | | | |
| **95% Confidence Interval** | | | | | | | |
|  |  | *Estimate* | *SE* | *t* | *p* | *Lower* | *Upper* |
| Phonological Fluency -> RMET | | 0.240 | 0.060 | 4.001 | **<0.001** | **0.120** | **0.361** |
| *Indirect Effects* | | | | | | | |
| **95% Confidence Interval** | | | | | | | |
|  |  | *Estimate* | | *BootSE* | | *BootLower* | *BootUpper* |
| Total |  | 0.081 | | 0.036 | | **0.010** | **0.153** |
| Phonological Fluency -> Language -> RMET | | 0.041 | | 0.029 | | -0.008 | 0.104 |
| Phonological Fluency > Memory -> RMET | | 0.030 | | 0.022 | | -0.007 | 0.079 |
| Phonological Fluency -> Visuospatial -> RMET | | 0.010 | | 0.023 | | -0.030 | 0.063 |
| *Total Effect* |  |  |  |  | |  |  |
| **95% Confidence Interval** | | | | | | | |
|  |  | *Estimate* | *SE* | *t* | *p* | *Lower* | *Upper* |
| Phonological Fluency -> RMET | | 0.322 | 0.060 | 5.368 | **<0.001** | **0.201** | **0.442** |
