# Supplementary material for: Theory of mind in mild cognitive impairment and Parkinson’s disease: The role of memory impairment
Source: Cogn Affect Behav Neurosci. 2023 Dec 4;24(1):156–70. doi: 10.3758/s13415-023-01142-z (PMC10827829; doi:10.3758/s13415-023-01142-z)
Supplement: Supplementary file 3 — Supplementary file3 (DOCX 26 KB) [file 13415_2023_1142_MOESM3_ESM.docx]

**Supplementary Material 3.**

| ***Predictor*** | | | | | | | |
| --- | --- | --- | --- | --- | --- | --- | --- |
| Color Reading – Stroop Test | | | | | | | |
| *Direct Effect* | | | | | | | |
| **95% Confidence Interval** | | | | | | | |
|  |  | *Estimate* | *SE* | *t* | *p* | *Lower* | *Upper* |
| Color Reading -> EAT | | 0.106 | 0.049 | 2.169 | **0.035** | **0.008** | **0.204** |
| *Indirect Effects* | | | | | | | |
| **95% Confidence Interval** | | | | | | | |
|  |  | *Estimate* | | *BootSE* | | *BootLower* | *BootUpper* |
| Total |  | 0.059 | | 0.032 | | -0.008 | 0.119 |
| Color Reading -> Language -> EAT | | 0.019 | | 0.018 | | -0.013 | 0.057 |
| Color Reading -> Memory -> EAT | | 0.047 | | 0.022 | | **0.007** | **0.094** |
| Color Reading -> Visuospatial -> EAT | | -0.007 | | 0.022 | | -0.057 | 0.035 |
| *Total Effect* |  |  |  |  | |  |  |
| **95% Confidence Interval** | | | | | | | |
|  |  | *Estimate* | *SE* | *t* | *p* | *Lower* | *Upper* |
| Color Reading -> EAT | | 0.165 | 0.047 | 3.521 | **<0.001** | **0.071** | **0.259** |

Mediation Models with Emotion Attribution Task (EAT) as outcome and single executive functions test as independent variable.

| ***Predictor*** | | | | | | | |
| --- | --- | --- | --- | --- | --- | --- | --- |
| Interference task – Stroop Test | | | | | | | |
| *Direct Effect* | | | | | | | |
| **95% Confidence Interval** | | | | | | | |
|  |  | *Estimate* | *SE* | *t* | *p* | *Lower* | *Upper* |
| Interference task -> EAT | | 0.130 | 0.076 | 1.719 | 0.092 | -0.022 | 0.283 |
| *Indirect Effects* | | | | | | | |
| **95% Confidence Interval** | | | | | | | |
|  |  | *Estimate* | | *BootSE* | | *BootLower* | *BootUpper* |
| Total |  | 0.092 | | 0.049 | | **0.005** | **0.193** |
| Interference task -> Language -> EAT | | 0.038 | | 0.026 | | -0.009 | 0.096 |
| Interference task -> Memory -> EAT | | 0.071 | | 0.033 | | **0.015** | **0.147** |
| Interference task -> Visuospatial -> EAT | | -0.017 | | 0.040 | | -0.102 | 0.059 |
| *Total Effect* |  |  |  |  | |  |  |
| **95% Confidence Interval** | | | | | | | |
|  |  | *Estimate* | *SE* | *t* | *p* | *Lower* | *Upper* |
| Interference task -> EAT | | 0.222 | 0.070 | 3.192 | **0.002** | **0.083** | **0.362** |

| ***Predictor*** | | | | | | | |
| --- | --- | --- | --- | --- | --- | --- | --- |
| Trail Making Test part A (TMT:A) | | | | | | | |
| *Direct Effect* | | | | | | | |
| **95% Confidence Interval** | | | | | | | |
|  |  | *Estimate* | *SE* | *t* | *p* | *Lower* | *Upper* |
| TMT:A -> EAT | | -0.016 | 0.012 | -1.312 | 0.196 | -0.040 | 0.008 |
| *Indirect Effects* | | | | | | | |
| **95% Confidence Interval** | | | | | | | |
|  |  | *Estimate* | | *BootSE* | | *BootLower* | *BootUpper* |
| Total |  | -0.015 | | 0.008 | | -0.032 | 0.001 |
| TMT:A -> Language -> EAT | | -0.006 | | 0.005 | | -0.019 | 0.001 |
| TMT:A -> Memory -> EAT | | -0.011 | | 0.005 | | **-0.022** | **-0.003** |
| TMT:A -> Visuospatial -> EAT | | 0.002 | | 0.006 | | -0.009 | 0.017 |
| *Total Effect* |  |  |  |  | |  |  |
| **95% Confidence Interval** | | | | | | | |
|  |  | *Estimate* | *SE* | *t* | *p* | *Lower* | *Upper* |
| TMT:A -> EAT | | -0.030 | 0.012 | -2.737 | **0.008** | **-0.053** | **-0.008** |

| ***Predictor*** | | | | | | | |
| --- | --- | --- | --- | --- | --- | --- | --- |
| Trail Making Test B-A (TMT:B-A) | | | | | | | |
| *Direct Effect* | | | | | | | |
| **95% Confidence Interval** | | | | | | | |
|  |  | *Estimate* | *SE* | *t* | *p* | *Lower* | *Upper* |
| TMT:B-A -> EAT | | -0.016 | 0.005 | -3.362 | **0.001** | **-0.026** | **-0.006** |
| *Indirect Effects* | | | | | | | |
| **95% Confidence Interval** | | | | | | | |
|  |  | *Estimate* | | *BootSE* | | *BootLower* | *BootUpper* |
| Total |  | -0.004 | | 0.003 | | -0.010 | 0.002 |
| TMT:B-A -> Language -> EAT | | -0.002 | | 0.002 | | -0.006 | 0.001 |
| TMT:B-A -> Memory -> EAT | | -0.004 | | 0.002 | | **-0.008** | **-0.001** |
| TMT:B-A -> Visuospatial -> EAT | | 0.002 | | 0.003 | | -0.003 | 0.008 |
| *Total Effect* |  |  |  |  | |  |  |
| **95% Confidence Interval** | | | | | | | |
|  |  | *Estimate* | *SE* | *t* | *p* | *Lower* | *Upper* |
| TMT:B-A -> EAT | | -0.020 | 0.005 | -4.436 | **<0.001** | **-0.029** | **-0.011** |

| ***Predictor*** | | | | | | | |
| --- | --- | --- | --- | --- | --- | --- | --- |
| Phonological Fluency | | | | | | | |
| *Direct Effect* | | | | | | | |
| **95% Confidence Interval** | | | | | | | |
|  |  | *Estimate* | *SE* | *t* | *p* | *Lower* | *Upper* |
| Phonological Fluency -> EAT | | 0.159 | 0.057 | 2.796 | **0.007** | **0.045** | **0.273** |
| *Indirect Effects* | | | | | | | |
| **95% Confidence Interval** | | | | | | | |
|  |  | *Estimate* | | *BootSE* | | *BootLower* | *BootUpper* |
| Total |  | 0.067 | | 0.031 | | **0.008** | **0.134** |
| Phonological Fluency -> Language -> EAT | | 0.024 | | 0.021 | | -0.008 | 0.074 |
| Phonological Fluency > Memory -> EAT | | 0.047 | | 0.023 | | **0.007** | **0.096** |
| Phonological Fluency -> Visuospatial -> EAT | | -0.004 | | 0.020 | | -0.047 | 0.038 |
| *Total Effect* |  |  |  |  | |  |  |
| **95% Confidence Interval** | | | | | | | |
|  |  | *Estimate* | *SE* | *t* | *p* | *Lower* | *Upper* |
| Phonological Fluency -> EAT | | 0.226 | 0.056 | 4.071 | **<0.001** | **0.115** | **0.338** |
