# Supplementary material for: Theory of mind in mild cognitive impairment and Parkinson’s disease: The role of memory impairment
Source: Cogn Affect Behav Neurosci. 2023 Dec 4;24(1):156–70. doi: 10.3758/s13415-023-01142-z (PMC10827829; doi:10.3758/s13415-023-01142-z)
Supplement: Supplementary file 4 — Supplementary file4 (DOCX 26 KB) [file 13415_2023_1142_MOESM4_ESM.docx]

**Supplementary Material 4.**

| ***Predictor*** | | | | | | | |
| --- | --- | --- | --- | --- | --- | --- | --- |
| Color Reading – Stroop Test | | | | | | | |
| *Direct Effect* | | | | | | | |
| **95% Confidence Interval** | | | | | | | |
|  |  | *Estimate* | *SE* | *t* | *p* | *Lower* | *Upper* |
| Color Reading -> ATT | | -0.008 | 0.032 | -0.260 | 0.796 | -0.073 | 0.056 |
| *Indirect Effects* | | | | | | | |
| **95% Confidence Interval** | | | | | | | |
|  |  | *Estimate* | | *BootSE* | | *BootLower* | *BootUpper* |
| Total |  | 0.046 | | 0.019 | | **0.012** | **0.088** |
| Color Reading -> Language -> ATT | | 0.006 | | 0.009 | | -0.009 | 0.027 |
| Color Reading -> Memory -> ATT | | 0.022 | | 0.018 | | -0.006 | 0.063 |
| Color Reading -> Visuospatial -> ATT | | 0.018 | | 0.016 | | -0.011 | 0.054 |
| *Total Effect* |  |  |  |  | |  |  |
| **95% Confidence Interval** | | | | | | | |
|  |  | *Estimate* | *SE* | *t* | *p* | *Lower* | *Upper* |
| Color Reading -> ATT | | 0.037 | 0.030 | 1.246 | 0.219 | -0.023 | 0.097 |

Mediation Models with Strange Stories (ATT) as outcome and single executive functions test as independent variable.

| ***Predictor*** | | | | | | | |
| --- | --- | --- | --- | --- | --- | --- | --- |
| Interference task – Stroop Test | | | | | | | |
| *Direct Effect* | | | | | | | |
| **95% Confidence Interval** | | | | | | | |
|  |  | *Estimate* | *SE* | *t* | *p* | *Lower* | *Upper* |
| Interference task -> ATT | | 0.001 | 0.048 | 0.025 | 0.980 | -0.096 | 0.098 |
| *Indirect Effects* | | | | | | | |
| **95% Confidence Interval** | | | | | | | |
|  |  | *Estimate* | | *BootSE* | | *BootLower* | *BootUpper* |
| Total |  | 0.071 | | 0.032 | | **0.013** | **0.140** |
| Interference task -> Language -> ATT | | 0.013 | | 0.013 | | -0.015 | 0.039 |
| Interference task -> Memory -> ATT | | 0.028 | | 0.025 | | -0.011 | 0.087 |
| Interference task -> Visuospatial -> ATT | | 0.029 | | 0.029 | | -0.026 | 0.089 |
| *Total Effect* |  |  |  |  | |  |  |
| **95% Confidence Interval** | | | | | | | |
|  |  | *Estimate* | *SE* | *t* | *p* | *Lower* | *Upper* |
| Interference task -> ATT | | 0.072 | 0.043 | 1.680 | 0.099 | -0.014 | 0.158 |

| ***Predictor*** | | | | | | | |
| --- | --- | --- | --- | --- | --- | --- | --- |
| Trail Making Test part A (TMT:A) | | | | | | | |
| *Direct Effect* | | | | | | | |
| **95% Confidence Interval** | | | | | | | |
|  |  | *Estimate* | *SE* | *t* | *p* | *Lower* | *Upper* |
| TMT:A -> ATT | | -0.007 | 0.007 | -0.954 | 0.345 | -0.021 | 0.008 |
| *Indirect Effects* | | | | | | | |
| **95% Confidence Interval** | | | | | | | |
|  |  | *Estimate* | | *BootSE* | | *BootLower* | *BootUpper* |
| Total |  | -0.009 | | 0.004 | | **-0.019** | **-0.002** |
| TMT:A -> Language -> ATT | | -0.002 | | 0.003 | | -0.008 | 0.002 |
| TMT:A -> Memory -> ATT | | -0.004 | | 0.003 | | -0.011 | 0.002 |
| TMT:A -> Visuospatial -> ATT | | -0.003 | | 0.004 | | -0.012 | 0.003 |
| *Total Effect* |  |  |  |  | |  |  |
| **95% Confidence Interval** | | | | | | | |
|  |  | *Estimate* | *SE* | *t* | *p* | *Lower* | *Upper* |
| TMT:A -> ATT | | -0.016 | 0.006 | -2.445 | **0.018** | **-0.028** | **-0.003** |

| ***Predictor*** | | | | | | | |
| --- | --- | --- | --- | --- | --- | --- | --- |
| Trail Making Test B-A (TMT:B-A) | | | | | | | |
| *Direct Effect* | | | | | | | |
| **95% Confidence Interval** | | | | | | | |
|  |  | *Estimate* | *SE* | *t* | *p* | *Lower* | *Upper* |
| TMT:B-A -> ATT | | -0.006 | 0.003 | -1.965 | 0.055 | -0.013 | 0.001 |
| *Indirect Effects* | | | | | | | |
| **95% Confidence Interval** | | | | | | | |
|  |  | *Estimate* | | *BootSE* | | *BootLower* | *BootUpper* |
| Total |  | -0.003 | | 0.002 | | -0.006 | 0.001 |
| TMT:B-A -> Language -> ATT | | -0.001 | | 0.001 | | -0.003 | 0.001 |
| TMT:B-A -> Memory -> ATT | | -0.001 | | 0.001 | | -0.004 | 0.001 |
| TMT:B-A -> Visuospatial -> ATT | | -0.001 | | 0.002 | | -0.005 | 0.003 |
| *Total Effect* |  |  |  |  | |  |  |
| **95% Confidence Interval** | | | | | | | |
|  |  | *Estimate* | *SE* | *t* | *p* | *Lower* | *Upper* |
| TMT:B-A -> ATT | | -0.009 | 0.003 | -3.184 | **0.002** | **-0.015** | **-0.003** |

| ***Predictor*** | | | | | | | |
| --- | --- | --- | --- | --- | --- | --- | --- |
| Phonological Fluency | | | | | | | |
| *Direct Effect* | | | | | | | |
| **95% Confidence Interval** | | | | | | | |
|  |  | *Estimate* | *SE* | *t* | *p* | *Lower* | *Upper* |
| Phonological Fluency -> ATT | | 0.071 | 0.036 | 1.982 | 0.053 | -0.001 | 0.142 |
| *Indirect Effects* | | | | | | | |
| **95% Confidence Interval** | | | | | | | |
|  |  | *Estimate* | | *BootSE* | | *BootLower* | *BootUpper* |
| Total |  | 0.034 | | 0.018 | | -0.001 | 0.068 |
| Phonological Fluency -> Language -> ATT | | 0.006 | | 0.010 | | -0.011 | 0.030 |
| Phonological Fluency > Memory -> ATT | | 0.015 | | 0.016 | | -0.018 | 0.046 |
| Phonological Fluency -> Visuospatial -> ATT | | 0.013 | | 0.014 | | -0.014 | 0.044 |
| *Total Effect* |  |  |  |  | |  |  |
| **95% Confidence Interval** | | | | | | | |
|  |  | *Estimate* | *SE* | *t* | *p* | *Lower* | *Upper* |
| Phonological Fluency -> ATT | | 0.105 | 0.033 | 3.125 | **0.003** | **0.037** | **0.172** |
