# Supplementary material for: Theory of mind in mild cognitive impairment and Parkinson’s disease: The role of memory impairment
Source: Cogn Affect Behav Neurosci. 2023 Dec 4;24(1):156–70. doi: 10.3758/s13415-023-01142-z (PMC10827829; doi:10.3758/s13415-023-01142-z)
Supplement: Supplementary file 5 — Supplementary file5 (DOCX 27 KB) [file 13415_2023_1142_MOESM5_ESM.docx]

**Supplementary Material 5.**

| ***Predictor*** | | | | | | | |
| --- | --- | --- | --- | --- | --- | --- | --- |
| Color Reading – Stroop Test | | | | | | | |
| *Direct Effect* | | | | | | | |
| **95% Confidence Interval** | | | | | | | |
|  |  | *Estimate* | *SE* | *t* | *p* | *Lower* | *Upper* |
| Color Reading -> TMPS | | 0.213 | 0.103 | 2.070 | **0.044** | **0.006** | **0.420** |
| *Indirect Effects* | | | | | | | |
| **95% Confidence Interval** | | | | | | | |
|  |  | *Estimate* | | *BootSE* | | *BootLower* | *BootUpper* |
| Total |  | 0.297 | | 0.090 | | **0.124** | **0.472** |
| Color Reading -> Language -> TMPS | | 0.069 | | 0.053 | | -0.030 | 0.179 |
| Color Reading -> Memory -> TMPS | | 0.199 | | 0.076 | | **0.072** | **0.371** |
| Color Reading -> Visuospatial -> TMPS | | 0.029 | | 0.043 | | -0.057 | 0.117 |
| *Total Effect* |  |  |  |  | |  |  |
| **95% Confidence Interval** | | | | | | | |
|  |  | *Estimate* | *SE* | *t* | *p* | *Lower* | *Upper* |
| Color Reading -> TMPS | | 0.511 | 0.124 | 4.121 | **<0.001** | **0.262** | **0.759** |

Mediation Models with Theory of Mind Picture Stories Task (TMPS) as outcome and single executive functions test as independent variable.

| ***Predictor*** | | | | | | | |
| --- | --- | --- | --- | --- | --- | --- | --- |
| Interference task – Stroop Test | | | | | | | |
| *Direct Effect* | | | | | | | |
| **95% Confidence Interval** | | | | | | | |
|  |  | *Estimate* | *SE* | *t* | *p* | *Lower* | *Upper* |
| Interference task -> TMPS | | 0.551 | 0.143 | 3.837 | **<0.001** | **0.262** | **0.839** |
| *Indirect Effects* | | | | | | | |
| **95% Confidence Interval** | | | | | | | |
|  |  | *Estimate* | | *BootSE* | | *BootLower* | *BootUpper* |
| Total |  | 0.399 | | 0.141 | | **0.143** | **0.696** |
| Interference task -> Language -> TMPS | | 0.121 | | 0.060 | | **0.022** | **0.258** |
| Interference task -> Memory -> TMPS | | 0.268 | | 0.110 | | **0.080** | **0.504** |
| Interference task -> Visuospatial -> TMPS | | 0.009 | | 0.071 | | -0.138 | 0.153 |
| *Total Effect* |  |  |  |  | |  |  |
| **95% Confidence Interval** | | | | | | | |
|  |  | *Estimate* | *SE* | *t* | *p* | *Lower* | *Upper* |
| Interference task -> TMPS | | 0.949 | 0.162 | 5.858 | **<0.001** | **0.624** | **1.274** |

| ***Predictor*** | | | | | | | |
| --- | --- | --- | --- | --- | --- | --- | --- |
| Trail Making Test part A (TMT:A) | | | | | | | |
| *Direct Effect* | | | | | | | |
| **95% Confidence Interval** | | | | | | | |
|  |  | *Estimate* | *SE* | *t* | *p* | *Lower* | *Upper* |
| TMT:A -> TMPS | | -0.078 | 0.023 | -3.381 | **0.001** | **-0.125** | **-0.032** |
| *Indirect Effects* | | | | | | | |
| **95% Confidence Interval** | | | | | | | |
|  |  | *Estimate* | | *BootSE* | | *BootLower* | *BootUpper* |
| Total |  | -0.061 | | 0.023 | | **-0.115** | **-0.021** |
| TMT:A -> Language -> TMPS | | -0.019 | | 0.013 | | **-0.055** | **-0.003** |
| TMT:A -> Memory -> TMPS | | -0.041 | | 0.015 | | **-0.073** | **-0.013** |
| TMT:A -> Visuospatial -> TMPS | | -0.001 | | 0.012 | | -0.026 | 0.023 |
| *Total Effect* |  |  |  |  | |  |  |
| **95% Confidence Interval** | | | | | | | |
|  |  | *Estimate* | *SE* | *t* | *p* | *Lower* | *Upper* |
| TMT:A -> TMPS | | -0.140 | 0.027 | -5.257 | **<0.001** | **-0.193** | **-0.086** |

| ***Predictor*** | | | | | | | |
| --- | --- | --- | --- | --- | --- | --- | --- |
| Trail Making Test B-A (TMT:B-A) | | | | | | | |
| *Direct Effect* | | | | | | | |
| **95% Confidence Interval** | | | | | | | |
|  |  | *Estimate* | *SE* | *t* | *p* | *Lower* | *Upper* |
| TMT:B-A -> TMPS | | -0.033 | 0.010 | -3.274 | **0.002** | **-0.053** | **-0.013** |
| *Indirect Effects* | | | | | | | |
| **95% Confidence Interval** | | | | | | | |
|  |  | *Estimate* | | *BootSE* | | *BootLower* | *BootUpper* |
| Total |  | -0.024 | | 0.010 | | **-0.045** | **-0.007** |
| TMT:B-A -> Language -> TMPS | | -0.008 | | 0.005 | | **-0.018** | **-0.001** |
| TMT:B-A -> Memory -> TMPS | | -0.016 | | 0.007 | | **-0.032** | **-0.003** |
| TMT:B-A -> Visuospatial -> TMPS | | -0.001 | | 0.005 | | -0.010 | 0.009 |
| *Total Effect* |  |  |  |  | |  |  |
| **95% Confidence Interval** | | | | | | | |
|  |  | *Estimate* | *SE* | *t* | *p* | *Lower* | *Upper* |
| TMT:B-A -> TMPS | | -0.057 | 0.012 | -4.858 | **<0.001** | **-0.081** | **-0.033** |

| ***Predictor*** | | | | | | | |
| --- | --- | --- | --- | --- | --- | --- | --- |
| Phonological Fluency | | | | | | | |
| *Direct Effect* | | | | | | | |
| **95% Confidence Interval** | | | | | | | |
|  |  | *Estimate* | *SE* | *t* | *p* | *Lower* | *Upper* |
| Phonological Fluency -> TMPS | | 0.356 | 0.119 | 3.001 | **0.004** | **0.118** | **0.594** |
| *Indirect Effects* | | | | | | | |
| **95% Confidence Interval** | | | | | | | |
|  |  | *Estimate* | | *BootSE* | | *BootLower* | *BootUpper* |
| Total |  | 0.323 | | 0.111 | | **0.099** | **0.536** |
| Phonological Fluency -> Language -> TMPS | | 0.089 | | 0.061 | | -0.021 | 0.221 |
| Phonological Fluency > Memory -> TMPS | | 0.200 | | 0.081 | | **0.050** | **0.365** |
| Phonological Fluency -> Visuospatial -> TMPS | | 0.033 | | 0.044 | | -0.049 | 0.134 |
| *Total Effect* |  |  |  |  | |  |  |
| **95% Confidence Interval** | | | | | | | |
|  |  | *Estimate* | *SE* | *t* | *p* | *Lower* | *Upper* |
| Phonological Fluency -> TMPS | | 0.679 | 0.149 | 4.566 | **<0.001** | **0.380** | **0.977** |
